# Fine mapping of the *Chilli veinal mottle virus* resistance 4 (*cvr4*) gene in pepper (*Capsicum annuum* L.)

**DOI:** 10.1007/s00122-024-04805-8

**Published:** 2025-01-07

**Authors:** Joung-Ho Lee, Jung-Min Kim, Jin-Kyung Kwon, Byoung-Cheorl Kang

**Affiliations:** 1 Department of Agriculture, Forestry and Bioresources, Research Institute of Agriculture and Life Sciences, Plant Genomics and Breeding Institute, College of Agriculture and Life Sciences, Seoul National University, Seoul, 08826 Republic of Korea; 2 Interdisciplinary Program in Agricultural Biotechnology, College of Agriculture and Life Sciences, Seoul National University, Seoul, 08826 Republic of Korea; 3 FarmyirehSe Co., Ltd., Seoul, 08826 Republic of Korea

## Abstract

**Key message:**

The single recessive Chilli veinal mottle virus resistance locus, *cvr4*, was fine-mapped in pepper through bulked segregant RNA sequencing combined with gene silencing analysis.

**Abstract:**

Chilli veinal mottle virus (ChiVMV) is a widespread pathogen affecting the production of peppers (*Capsicum annuum* L.) in Asia and Africa. Few loci conferring resistance to ChiVMV have been identified, severely limiting the development of resistant cultivars. To identify ChiVMV resistance genes, we constructed an F_2:3_ segregating population derived from a cross between the ChiVMV-resistant cultivar ‘CV9’ and the susceptible cultivar ‘Jeju’. The inheritance study of F_2:3_ populations showed a 1:3 ratio of resistant to susceptible individuals, demonstrating the existence of a single recessive ChiVMV resistance gene in CV9; we named this gene *cvr4*. To map the *cvr4* locus, we employed bulked segregant analysis by RNA sequencing (BSR-seq) of pools from resistant and susceptible F_2:3_ individuals. We mapped *cvr4* to the telomeric region of pepper chromosome 11. To narrow down the *cvr4* locus, we developed additional molecular markers in the *cvr4* target region, leading to a 2-Mb region of chromosome 11 showing complete co-segregation with the ChiVMV resistance phenotype. Using the polymorphisms identified during BSR-seq, we defined a list of 15 candidate genes for *cvr4*, which we tested through virus-induced gene silencing analysis for ChiVMV resistance. Of these, the silencing of several genes (DEM.v1.00021323, DEM.v1.00021336, and DEM.v1.00021337) restricted virus spread. Although DEM.v1.00021323 transcript levels were similar between the resistant and susceptible bulks, its alternative spliced isoforms differed in abundance, suggesting that the splicing variants of DEM.v1.00021323 might affect viral infection. These findings may facilitate the breeding of ChiVMV-resistant cultivars in pepper.

**Supplementary Information:**

The online version contains supplementary material available at 10.1007/s00122-024-04805-8.

## Introduction

Potyviruses such as Chilli veinal mottle virus (ChiVMV) threaten the production of pepper (*Capsicum annuum* L.) worldwide (Barka and Lee 2020; Hernández-Pérez et al. 2020; Parisi et al. 2020). Ten *potyvirus resistance* (*pvr*) genes have been described in pepper (Rezende et al. 2020), three of which (*pvr1*, *Pvr4*, and *pvr6*) have been cloned. *Pvr4* is a dominant allele that encodes a nucleotide-binding leucine-rich repeat domain and interacts with the RNA-dependent RNA polymerase of the potyviruses (Kim et al. 2015). *pvr1* and *pvr6* are recessive alleles encoding eukaryotic translation initiation factor 4E (eIF4E) and eukaryotic translation initiation factor iso 4E [eIF(iso)4E], respectively (Kang et al. 2005a; Ruffel et al. 2006). The viral genome-linked protein (VPg), a potyviral protein covalently associated with the 5′ end of the viral RNA during potyvirus infection, interacts with various host proteins (de Oliveira et al. 2019). Mutations in their encoding genes have the potential to disrupt the interaction between VPg and these host factors, conferring resistance to multiple potyviruses in host plants (Charron et al. 2008; Kang et al. 2005a). These loci may act as susceptibility (*S*) genes, which encode host factors that viral pathogens use to aid their replication.

The *pvr* gene-linked molecular markers were actively used to breed the potyvirus-resistant cultivar. For example, *pvr1* was widely used for Tobacco etch virus (TEV) resistance breeding in pepper, while *Pvr4* was widely used for Pepper mottle virus (PepMoV) resistance breeding in pepper. For ChiVMV, the simultaneous mutations in the *Pvr1* and *Pvr6* allele, *pvr1*^*2*^ with *pvr6*, could confer the resistance in pepper, but it could not clearly explain the resistance in the F_1_ hybrid of the *pvr1*^*2*^ and *pvr6* (Hwang et al. 2009). For this reason, it is important to find the complete resistance sources and develop molecular markers for ChiVMV resistance in pepper. Although many other genetic sources of ChiVMV resistance have been reported in pepper, most remain to be characterized (Barchenger et al. 2019). For example, several cultivars have been reported carrying dominant, recessive, or polygenic ChiVMV resistance genes (Caranta and Palloix 1996; Lee et al. 2013, 2017; Naresh et al. 2016). A resistance locus in the pepper cultivars ‘NW4’, ‘CV3’, and ‘CV8’ was identified as being the same gene (*ChiVMV resistance 1*, *Cvr1*) and was mapped to the short arm of chromosome 6 (Lee et al. 2013, 2017). Furthermore, a recessive ChiVMV resistance locus, *ChiVMVR9* (*ChiVMV Resistance 9*) from IHR2451, was mapped to pepper chromosome 9 (Ponnam et al. 2023). Nevertheless, the polygenic and the other single recessive resistance loci in ‘CV4’ and ‘CV9’ have not yet been mapped (Lee et al. 2017).

In this study, we applied bulked segregant RNA-seq analysis (BSR-seq) (Koeda et al. 2021; Solomon et al. 2021) to fine-map the single recessive ChiVMV resistance locus *cvr4* (*Chilli veinal mottle virus resistance 4*) and identified 15 candidate genes. Virus-induced gene silencing (VIGS) of each candidate highlighted one candidate gene for *cvr4*, which encodes a protein associated with ribosome biogenesis. These mapping results may expedite the development of ChiVMV-resistant cultivars and offer insights into the mechanism of ChiVMV resistance in plants.

## Materials and methods

### Plant materials

The ChiVMV-resistant pepper (*C. annuum*) accession CV9 was obtained from Clover Seed Ltd. (Shouson Hill, Hong Kong). An F_2_ population was generated by crossing the ChiVMV-susceptible cultivar ‘Jeju’ to CV9. F_2_ individuals derived from this cross were self-pollinated to generate an F_2:3_ population. To assess resistance to ChiVMV, at least 24 F_2:3_ individuals were inoculated with the virus. The plants were cultivated in a growth chamber at 23 °C and under a photoperiod of 16 h light/8 h dark. To evaluate potyvirus screening, *C. annuum* ‘CM334’ (*Pvr4*/*Pvr4*) was used as the resistant accession against PepMoV-Vb1 infection (Kim et al. 2017a, b), and *C. chinense* ‘PI152225’ (*pvr1*/*pvr1*) was used as the resistant accession against TEV-HAT infection (Venkatesh et al. 2018). Jeju (*C. annuum*) was used as the susceptible accession for all potyvirus infections.

### Evaluation of potyvirus resistance

Inocula were freshly propagated in *Nicotiana benthamiana* before screening the CV9 × Jeju F_2:3_ population. ChiVMV was initially obtained from ChiVMV-infected pepper plants in the experimental station of Nongwoo Bio in Indonesia (Hwang et al. 2009). Then, ChiVMV inoculum was propagated twice from the first ChiVMV stock to minimize the risk of mutations in *N. benthamiana*. To maintain an infectious stock of ChiVMV, an inoculum in potassium phosphate buffer (pH 7.0) was mechanically inoculated onto *N. benthamiana* leaves by carborundum dusting (SPCCOK, Busan, Korea). The infectious stock of ChiVMV was stored at -80 °C. All pepper plants were mechanically inoculated with fresh ChiVMV inocula (2 g of ChiVMV inocula/10 mL of potassium phosphate buffer) using carborundum as previously described (Hwang et al. 2009). Before pepper screening, we recovered the frozen ChiVMV stock by inoculating it into *N. benthamiana* again. The freshly ChiVMV-infected *N. benthamiana* leaves were then used for the pepper screening. The same method was followed for inoculation with two other potyviruses, TEV-HAT and PepMoV. To detect the coat protein (CP) of viruses, a double antibody sandwich enzyme-linked immunosorbent assay (DAS-ELISA) was performed according to the manufacturer’s protocol (Agdia, Elkhart, IN, USA). The absorbance value at 405 nm was observed using a microplate reader (Anthon zenith 340 microplate reader, UK).

### Nucleic acid extraction and polymerase chain reaction (PCR)

Genomic DNA (gDNA) was extracted from young true leaves using a modified CTAB buffer protocol (Porebski et al. 1997). The DNA concentration was determined using an Epoch microplate reader (BioTek, VT, USA) and diluted to 50 ng/μL for genotyping analysis. The prepared gDNA was used as template DNA for PCR, high-resolution melting curve (HRM) analysis, or Kompetitive allele-specific PCR (KASP) assay (KASP, LGC Biosearch Technologies, Hoddesdon, UK). For PCR, the conditions were as follows: denaturation at 94 °C for 5 min, followed by 35 cycles of denaturation at 94 °C for 30 s, annealing at 58 °C for 30 s, and extension at 72 °C for 1 min, with one final cycle of post-extension at 72 °C for 10 min. For HRM analysis, the conditions were as follows: denaturation at 94 °C for 5 min, followed by 55 cycles of denaturation at 94 °C for 20 s, annealing at 58 °C for 20 s, and extension at 72 °C for 20 s, after which fluorescence was detected from 60 °C to 95 °C with a 0.1℃ increase. For the KASP assay, PCR mixtures were prepared using 100 ng of DNA. PCR was performed with the following conditions: 94 °C for 15 min; 10 cycles at 94 °C for 20 s and 61–55 °C for 1 min (decreasing 0.6 °C per cycle); 26 cycles at 94 °C for 20 s and 55 °C for 60 s. KASP genotyping assays were conducted using a LightCycler® 480 instrument II (Roche, Rotkreuz, Switzerland).

Total RNA was extracted from the leaves of various pepper plants using an MG Total RNA extraction kit (MGmed, Seoul, Korea) according to the manufacturer’s protocol. RNA concentration was determined using an Epoch microplate reader (BioTek, VT, USA). Two micrograms of total RNA were used in conjunction with 1 × ES reaction mixture, 10 mM dNTP, and 0.5 μg oligo(dT) primers for first-strand cDNA synthesis according to the manufacturer’s protocol (Transgen, China). Subsequently, 3 μL of cDNA was employed for PCR with the following components: 1 × HiPi Plus Reaction buffer, 10 mM dNTPs, 10 pmol of primer pairs, and Taq DNA polymerase.

### Bulked segregant RNA sequencing (BSR-seq) analysis

To perform BSR-seq, an equal amount of total RNA extracted from 34 F_3_ individuals (17 resistant and 17 susceptible) was pooled. A TruSeq RNA library prep kit v2 (Illumina, CA, USA) was used to construct the two RNA-seq libraries, and sequencing was performed at Macrogen (Seoul, Korea). The raw Illumina reads were aligned to the pepper reference genome ‘Dempsey’ (Lee et al. 2022), using STAR version 2.7.5a (Dobin et al. 2013). The QTL-seq analysis pipeline was employed using the aligned reads for the resistant and susceptible pools (Takagi et al. 2013). The polymorphism data from the alignment files were incorporated into the reference file using the internal scripts of the QTL-seq pipeline. Single nucleotide polymorphisms (SNPs) were extracted from the alignment files of the two pools and filtered using samtools (Danecek et al. 2021), and their distribution was plotted using internal Perl and R scripts from the QTL-seq pipeline. The SNP-index value for each pool was calculated based on the number of aligned reads to the reference genome. The difference in SNP-index value between the resistant and susceptible pools was defined as the Δ(SNP-index) value. SNPs were filtered using the minimum mismatch filter 4 and SNP depth 7 from the SNP calling program, Coval (Kosugi et al. 2013). Subsequently, the 4 Mb regions with 50 kb increments were selected for the sliding window approach for Δ(SNP-index).

To identify and quantify transcript isoforms, the two RNA-seq datasets corresponding to each pool were aligned against the Dempsey reference genome using hisat2.2.1, and the transcripts were assembled using stringtie2.0.6 (Kim et al. 2019; Kovaka et al. 2019). The alignment and transcriptome assembly pipelines followed a published procedure (Liu et al. 2022). Briefly, RNA-seq reads were aligned using hisat2 with default options; the resulting aligned reads were quantified using stringtie2 with the following parameters, ‘-m 200 -a 10 –conservative -g 50 -u’. Subsequently, transcript information for the R and S pool RNA-seq data was used to assemble the transcript using the ‘merge’ option in stringtie2 with the following parameters, ‘-m 200 -c 3’. After transcriptome assembly, Transcripts per million (TPM) values were obtained using stringtie2 against newly assembled transcript files.

### Virus-induced gene silencing (VIGS)

The Tobacco rattle virus 2 (TRV2) vector-based silencing construct was created using the ligation-independent cloning (LIC) method as previously described (Kim et al. 2017a, b). Partial coding sequences of the candidate genes from the cDNA of the susceptible cultivar, ranging from 200 to 400 bp, were amplified using LIC adapter primers. To avoid off-target effects, we designed the gene-specific fragments following the recommended regions from the SGN VIGS tool (Fernandez-Pozo et al. 2015) and confirmed the sequences of gene-specific fragments by the Sanger sequencing before cloning. Each PCR amplicon was purified using a DNA purification kit (CosmoGenetech, Seoul, Korea) and treated with T4 DNA polymerase (Enzymatics, MA, USA) in a reaction mixture containing 1 × blue buffer and 10 mM dATP. In parallel, the *Pst*I-digested pTRV2-LIC vector was treated with T4 DNA polymerase in a reaction mixture containing 5 × blue buffer and 10 mM dTTP. The reactions treated with T4 DNA polymerase were incubated at 22 °C for 30 min, followed by termination of reaction at 75 °C for 20 min. The treated PCR amplicon and vector were then mixed in a 1:3 (vector:insert) molar ratio. The mixture was incubated at room temperature for 30 min and then transformed into *E. coli* Trans5α-competent cells (TranGen, China). The plasmids were extracted from transformants using an AccuPrep Plasmid Mini Extraction Kit (Bioneer, Daejeon, Korea), and their inserts were subjected to Sanger sequencing (Macrogen, Seoul, Korea). Confirmed plasmids were introduced into Agrobacterium (*Agrobacterium tumefaciens*) strain GV3101 via electroporation at 2.0 kV. Two control plasmids, pTRV2::*PDS* and pTRV2::*GFP*, were generously provided by Prof. Doil Choi at Seoul National University.

Agrobacterium colonies carrying pTRV1, pTRV2::*GFP*, pTRV2::*PDS*, or pTRV2::*gene-of-interest* were cultured at 28 °C for 2 days in 5 mL of LB. The Agrobacterium cells were collected by centrifugation and resuspended in infiltration buffer (10 mM MES, 10 mM MgCl_2_, and 200 μM acetosyringone) to a final OD_600_ of 0.6. The cultures containing the pTRV1 and pTRV2 constructs were mixed in a 1:1 ratio. The mixed cell suspensions were then incubated at room temperature for 3 h before infiltrating the abaxial side of both cotyledons from the susceptible cultivar, Jeju. The infiltrated seedlings were incubated at 16 °C in the dark for 1 day and then grown at 20 °C under a 16-h-light/8-h-dark photoperiod.

After 28 days post infiltration (dpi) of the TRV VIGS constructs, we confirmed the albino phenotypes on the 4–6 upper leaves from the pTRV::*PDS* infiltrated leaves. Then, we carefully inoculated the ChiVMV onto two leaves of the 4–6 upper leaves from the TRV-inoculated cotyledons. The inoculation method was same as that in pepper screening, but the 1 g of ChiVMV inocula/10 mL of potassium phosphate buffer was used to screen the VIGS plants.

### Phylogenetic analysis

To reconstruct the phylogenetic tree for the *cvr4* candidate gene, a BLASTp search was conducted using the *cvr4* protein sequence as a query, which yielded ortholog information in other pepper cultivars and species. Subsequently, the sequences for 11 *cvr4* homologs were obtained from the NCBI protein sequence database. The following sequences were included in the phylogenetic analysis: PHT67617.1 (*C. annuum* CM334), PHU02229.1 (*C. chinense* PI159236), PHT33660.1 (*C. baccatum* PBC81), XP_047255447.1 (*C. annuum* UCD-10X-F1), KAH0643386.1 (*Solanum tuberosum* Otava), XP_016434959.1 (*N. tabacum* TN90), XP_010320846.1 (*S. lycopersicum* Heinz 1706), XP_015076904.1 (*S. pennellii* LA0716), XP_027096915.1 (*Coffea arabica* Caturra red), and TYH23235.1 (*Gossypium darwinii* 1,808,015.09). The phylogenetic tree was reconstructed with MEGA 11 using the maximum-likelihood method (Tamura et al. 2021).

## Results

### Evaluation of potyvirus resistance in CV9

**Fig. 1:**
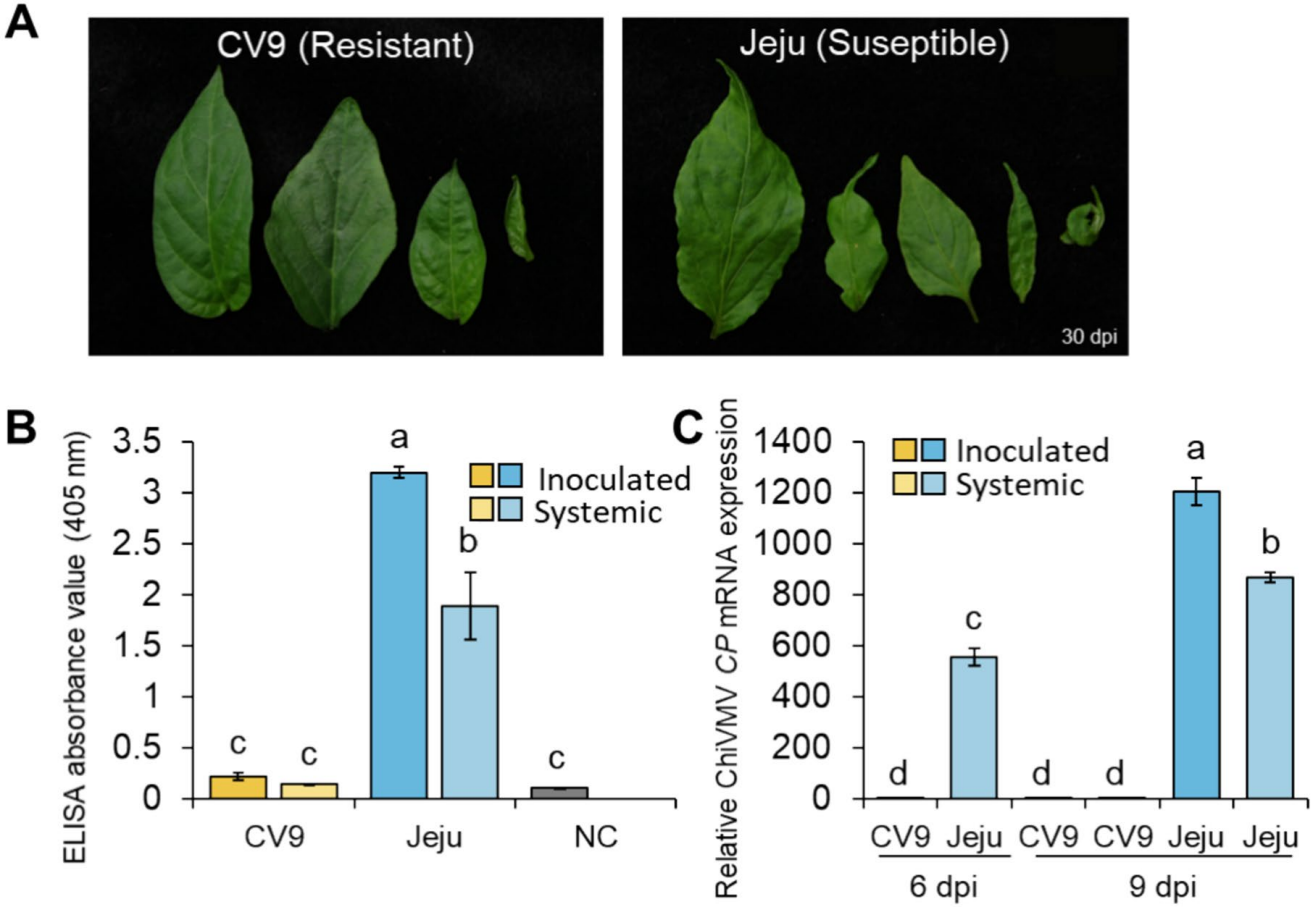
Evaluation of resistance to ChiVMV in the pepper accessions ‘CV9’ and ‘Jeju’. **A** Representative photographs showing ChiVMV symptoms in CV9 and Jeju at 30 days post inoculation (dpi). **B** Quantification of ChiVMV coat protein (CP) accumulation in inoculated and systemic leaves of CV9 and Jeju as detected by enzyme-linked immunosorbent assay (ELISA). Values are means ± standard error (SE) of the ELISA absorbance value (405 nm) for each accession (n = 10). NC refers to the negative controls, which are mock-inoculated leaves from Jeju. **C** Relative transcript levels of the ChiVMV *CP* gene in inoculated and systemic leaves of CV9 and Jeju. Values are means ± SE (n = 5). Different lowercase letters indicate significant differences, as determined by the Duncan’s multiple test with a *P*-value of at least 0.05

To confirm its resistance to ChiVMV, we inoculated CV9 plants with ChiVMV, along with the susceptible cultivar, Jeju. At 30 days post inoculation (dpi), we observed typical symptoms for ChiVMV, such as leaf mottling and leaf margin shrinkage, in Jeju but not in CV9 (Fig. 1A). An ELISA for the coat protein (CP) or ChiVMV detected very little viral CP in inoculated and systemic leaves of CV9 at 30 dpi, in contrast to the strong accumulation seen in Jeju (Fig. 1B). In agreement with the low abundance of the CP in CV9, the relative expression level of the ChiVMV *CP* gene was significantly higher in infected Jeju leaves compared to CV9 at 6 and 9 dpi (Fig. 1C). These results indicate that the resistant cultivar CV9 exhibits strong resistance to ChiVMV, blocking viral replication or cell-to-cell movement during ChiVMV infection.

**Fig. 2:**
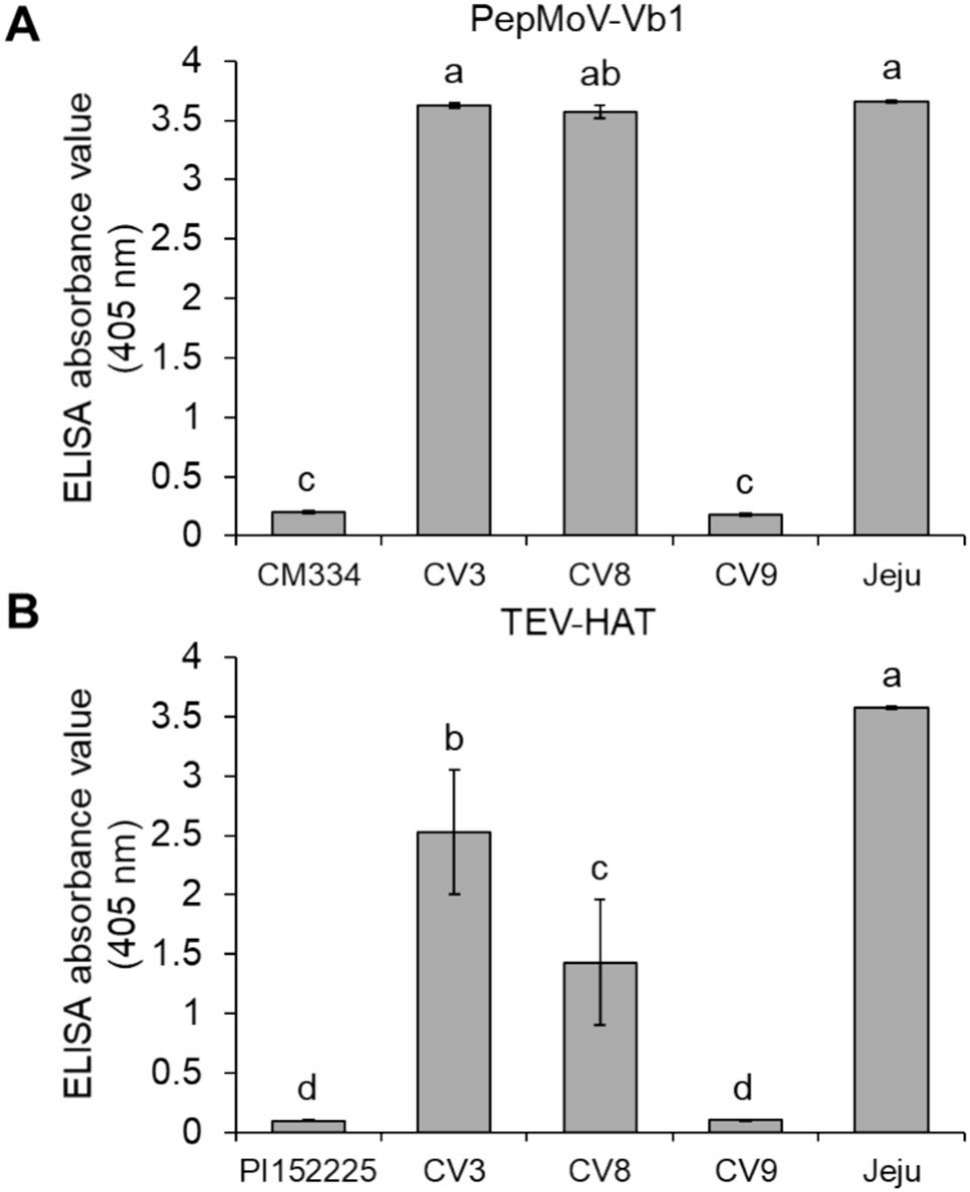
Test of potyvirus resistance spectrum in ChiVMV-resistant pepper accessions. **A** Assessment of resistance spectrum to PepMoV-Vb1 at 30 dpi, as determined by an ELISA for the accumulation of the CP. ‘CM334’ was used as an example of a resistant accession. **B** Assessment of resistance spectrum to TEV-HAT at 30 dpi, as determined by an ELISA for the accumulation of the CP. ‘PI152225’ was used as an example of a resistant accession. Values are means ± SE (n = 5). Different lowercase letters indicate significant differences, as determined by the Duncan’s multiple test with a *P*-value of at least 0.05

We also evaluated the spectrum of resistance to potyviruses in CV9 by challenging this accession with PepMoV-Vb1 and TEV-HAT, which are different species from ChiVMV but in the same genus, potyvirus. Accordingly, we inoculated CV9 plants with PepMoV-Vb1 or TEV-HAT and collected leaves at 30 dpi, for an ELISA using an antibody against the CP of PepMoV and TEV in each (Fig. 2). The ELISA signal for CV9 inoculated with either virus was significantly low similar to the resistant pepper accession CM334 (for PepMoV-Vb1) or PI152225 (for TEV-HAT). By contrast, Jeju plants inoculated with either virus showed a strong ELISA signal for the CP, indicating that this accession is generally susceptible to potyviruses. These results indicated that CV9 is an accession broadly resistant to potyviruses, not only ChiVMV but also PepMoV-Vb1 and TEV-HAT.

### Genetic analysis of ChiVMV resistance in CV9

We explored the inheritance pattern of ChiVMV resistance in CV9 by crossing CV9 to Jeju and evaluating virus resistance in their F_1_ hybrids and an F_2:3_ population. All F_1_ plants were susceptible, indicating that the resistance locus (or loci) in CV9 is (are) recessive. We also examined the resistance of F_2_ plants by screening their F_2:3_ progeny to help distinguish between F_2_ plants that are heterozygous for the CV9 allele or homozygous for the Jeju allele. The ratio of resistant to susceptible F_2_ individuals was 1:3 (44–127), indicating that the locus conferring resistance to ChiVMV in CV9 follows a single recessive inheritance pattern (Table 1). When we checked the resistance of the F_2:3_ progeny derived from susceptible F_2_ individuals, we determined that 86 F_2_ individuals were heterozygous for the CV9 allele, with the remaining 41 F_2_ individuals being homozygous for the Jeju allele, which supports the single recessive inheritance of ChiVMV resistance in CV9. We conclude that the resistance exhibited by CV9 toward ChiVMV is controlled by a single recessive locus, which we designated *cvr4*.

**Table 1:** Inheritance analysis of resistance to ChiVMV in F_1_ and F_2:3_ segregating populations derived from a cross between CV9 and Jeju

Genotype	Phenotype			*P*-value^*^
	Number of individuals	R	S	
CV9	10	10	0	NA
Jeju	10	0	10	NA
(CV9 x Jeju) F_1_	13	0	13	NA
(CV9 x Jeju) F_2:3_	171	44	127	0.945

^*^ *P*-value was calculated from the Chi-square test

R, resistant; S, susceptible; NA, not applicable

We asked whether the *cvr4* locus was distinct from previously reported recessive potyvirus resistance genes by genotyping the CV9 × Jeju F_2:3_ population for markers developed for the *pvr1*^*2*^ and *pvr6* genes (Hwang et al. 2009). Simultaneous mutations of *pvr1*^*2*^ with *pvr6* confer resistance to ChiVMV in *C. annuum* Perennial, but neither marker was co-segregated with the ChiVMV resistance phenotype in the CV9 × Jeju F_2:3_ population (Table S1). Furthermore, a sequence analysis of the *pvr1* and *pvr6* genes in CV9 and Jeju revealed that CV9 carries the *Pvr1*^+^ (dominant susceptible) and *pvr6* (recessive resistant) alleles, which have not previously been reported as ChiVMV-resistant alleles (Figure S1). This result suggests that the *cvr4* locus represents a previously unknown resistance locus for ChiVMV.

### BSR-seq analysis to identify the *cvr4* locus

To map the *cvr4* locus, we employed a BSR-seq approach. Specifically, we extracted 34 F_3_ RNAs (17 resistant and 17 susceptible) and pooled lines with a resistant or susceptible phenotype to ChiVMV infection, defining two bulk samples. We extracted total RNA from each bulk and constructed sequencing libraries, which were sequenced on a single Illumina sequencing lane.

We obtained 193 and 161 million sequences for the resistant and susceptible pools, respectively, with a sequencing depth of 996 × and 830 × for each bulk (Table S2). We aligned all RNA-seq reads to the pepper reference genome, Dempsey (Lee et al. 2022). The average number of reads uniquely mapped to Dempsey was 155 million (175 and 135 million to the resistant and susceptible pools in each), with 87% of the total number of reads. Using these uniquely mapped reads, we subsequently called SNPs and calculated the Δ(SNP-index) values using the QTL-seq pipeline (Takagi et al. 2013). We identified 22,355 SNPs through a QTL-seq analysis, with 14,682 SNPs later excluded due to the low quality of SNPs (Table S3). Subsequently, using the Dempsey genome as a physical reference for the positions of the 7,673 remaining SNPs, we constructed 40,728 sliding windows (4 Mb regions with 50 kb increments).

**Fig. 3:**
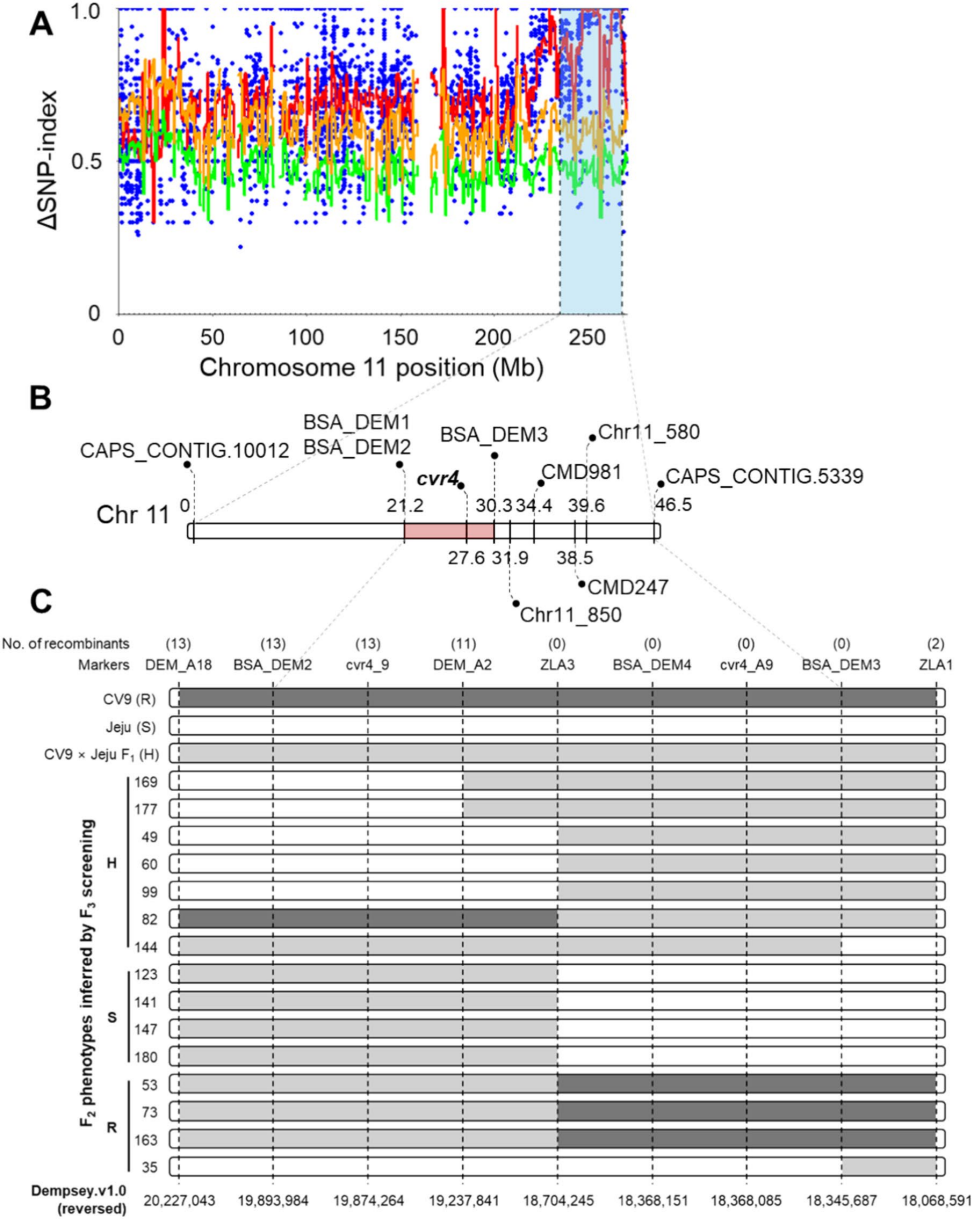
The *cvr4* locus maps to pepper chromosome 11. **A** Δ(SNP-index) plot calculated from the BSR-seq data for pepper chromosome 11. The red line indicates the average value of Δ(SNP-index) for each window (4 Mb size with 50 kb increment). The blue circles represent individual Δ(SNP-index) values at each chromosomal position. The green and orange lines indicate the 95% and 99% confidence intervals for Δ(SNP-index). **B** Partial genetic map of the *cvr4* locus on chromosome 11. Numbers of the diagram represent the genetic distance of markers, with their names shown. **C** Recombination map of markers closely linked to the *cvr4* locus. Plants homozygous for the CV9 or Jeju allele or heterozygous at the indicated markers are indicated by black, white and gray bars, respectively. Numbers in parentheses above the map represent the number of recombinants for each marker. The phenotypes of F_2_ individuals were inferred by screening F_2_-derived F_3_ individuals

We plotted the Δ(SNP-index) values across the genome using these filtered SNPs and sliding windows (Figure S2). We defined a window as being significant when the average Δ(SNP-index) value was higher than the 99% confidence interval value of the sliding window. We identified six windows reaching values above the 99% confidence interval, including windows on chromosomes 2, 7, 8, and 11, with the latter chromosome showing the highest peak of Δ(SNP-index) values (Fig. 3A**; **Figure S2; Table S4).

### Fine mapping of the *cvr4* locus

To confirm the position of the *cvr4* locus on chromosome 11, we genotyped the CV9 F_2:3_ population using two previously reported SNP markers, CAPS_CONTIG.10012 and CAPS_CONTIG.5339, which flank the region with the highest Δ(SNP-index) values on pepper chromosome 11 (Kang et al. 2014). Indeed, the two markers were linked to the *cvr4* locus, with a genetic distance from the *cvr4* locus of 27.6 cM (CAPS_CONTIG.10012) and 19.9 cM (CAPS_CONTIG.5339), respectively (Fig. 3B). To narrow down the location of the *cvr4* locus, we developed additional markers covering this region based on the SNP data obtained from the BSR-seq analysis (Table S5). We thus delineated the *cvr4* region to an ~ 2-Mb interval between the two markers, BSA_DEM2 and BSA_DEM3, spanning a region from 18.06 to 20.22 Mb in the reference genome ‘Dempsey’ (Fig. 3B).

In an attempt to fine-map the *cvr4* locus, we developed four complete co-segregating markers with *cvr4* (cvr4_A9, BSA_DEM4, ZLA3, and BSA_DEM3) (Fig. 3C). We tentatively located the *cvr4* candidate region between the two closely-linked markers ZLA1 (18.06 Mb) and DEM_A2 (19.23 Mb). Within this candidate region, the Dempsey genome carries 42 genes (Data S1). From the SNP calling data of the BSA RNA-seq results, we identified 95 SNPs in 15 out of 42 genes. We considered these genes as possible *cvr4* candidate genes (Table 2).

**Table 2:** List of the *cvr4* candidate genes and their read counts at 10 dpi with ChiVMV

Gene ID	Predicted function	Read counts	
		R	S
DEM.v1.00021387	Protein of unknown function	5137	4864
DEM.v1.00021374	Protein of unknown function	3068	2775
DEM.v1.00021373	Magnesium transporter MRS2-5	5078	3558
DEM.v1.00021372	Ninja-family protein AFP1	3874	2160
DEM.v1.00021360	Putative methylesterase 11	240	1132
DEM.v1.00021337	Probable 6-phosphogluconolactonase 1	2551	2968
DEM.v1.00021336	Fructokinase-like 2	8337	9817
DEM.v1.00021335	Folate synthesis bifunctional protein	1794	1668
DEM.v1.00021323	Nucleolar protein 14	14,311	12,618
DEM.v1.00021274	Protein of unknown function	10	15
DEM.v1.00021266	Cytochrome P450 CYP749A22	235	69
DEM.v1.00021265	Protein of unknown function	1783	2529
DEM.v1.00021264	ACT domain-containing protein ACR4	8071	5090
DEM.v1.00021263	Pentatricopeptide repeat-containing protein At1g10910	16,332	14,995
DEM.v1.00021258	SOT12 Cytosolic sulfotransferase 12	185	449

R, resistant; S, susceptible

### VIGS-mediated testing of the *cvr4* candidate genes

**Fig. 4:**
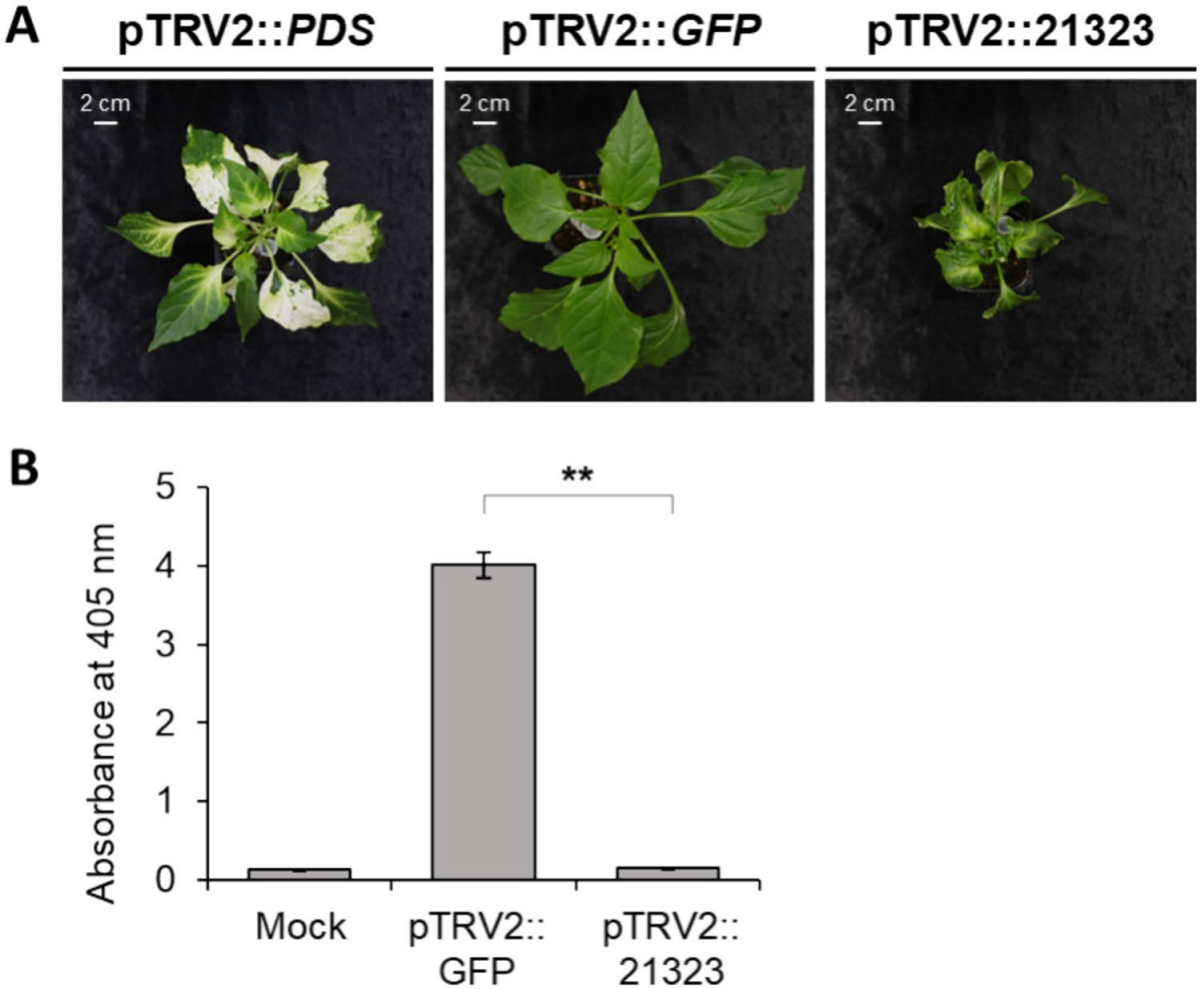
Assessing resistance to ChiVMV in the susceptible accession Jeju with VIGS for individual *cvr4* candidate genes. **A** Representative photographs of Jeju plants inoculated with the control VIGS constructs pTRV2::*PDS* or pTRV2::*GFP*, or with the VIGS construct silencing the *cvr4* candidate gene DEM0021323 (pTRV2::21,323). **B** Quantification of ChiVMV CP accumulation in the mock-inoculated Jeju plants (NC) or Jeju plants inoculated with pTRV2::*GFP* or pTRV2::*21,323* silencing the *cvr4* candidate gene, as determined by an ELISA. Asterisks indicate significant differences as determined by the Student’s t-test with a *P*-value < 0.05

To identify the *cvr4* gene in the target region, we employed a VIGS approach in the susceptible cultivar Jeju to observe the consequences of silencing each candidate gene on virus resistance. To this end, we cloned specific fragments for each of the 15 *cvr4* candidate genes into the pTRV2 vector and infiltrated the cotyledons of pepper seedlings with each construct. About 21 days after infiltration, we inoculated each silenced plant with ChiVMV (Figure S3). Of the 15 genes tested, we observed no significant decrease in the transcript levels for five genes (DEM.v1.00021258, DEM.v1.00021274, DEM.v1.00021360, DEM.v1.00021374, and DEM.v1.00021387) compared to seedlings infiltrated with a pTRV2::*GFP* control construct, for unknown reasons. Among the 10 remaining genes with effective VIGS-mediated silencing, the silencing of DEM.v1.00021323, DEM.v1.00021336, and DEM.v1.00021337 showed a significant drop in CP accumulation compared to the pTRV2::*GFP* plants infected with ChiVMV (Fig. 4A, B). Among them, the silencing of DEM.v1.00021323 was highly associated with a ChiVMV resistance in the silenced plants and also reduced the plant growth.

### Characterization of the *cvr4* candidate gene DEM.v1.00021323

We investigated the expression pattern of all *cvr4* candidate genes including DEM.v1.00021323 in CV9 and Jeju plants inoculated with ChiVMV (Figure S4). The expression level of DEM.v1.00021323 was not significantly different between CV9 and Jeju at any point before (0 h post inoculation [hpi]) and following inoculation. Its expression decreased in the early stage of infection (4–6 hpi), before gradually increasing. Two additional genes within the *cvr4* candidate region (DEM.v1.00021265 and DEM.v1.00021337) exhibited similar expression profiles during ChiVMV infection.

**Fig. 5:**
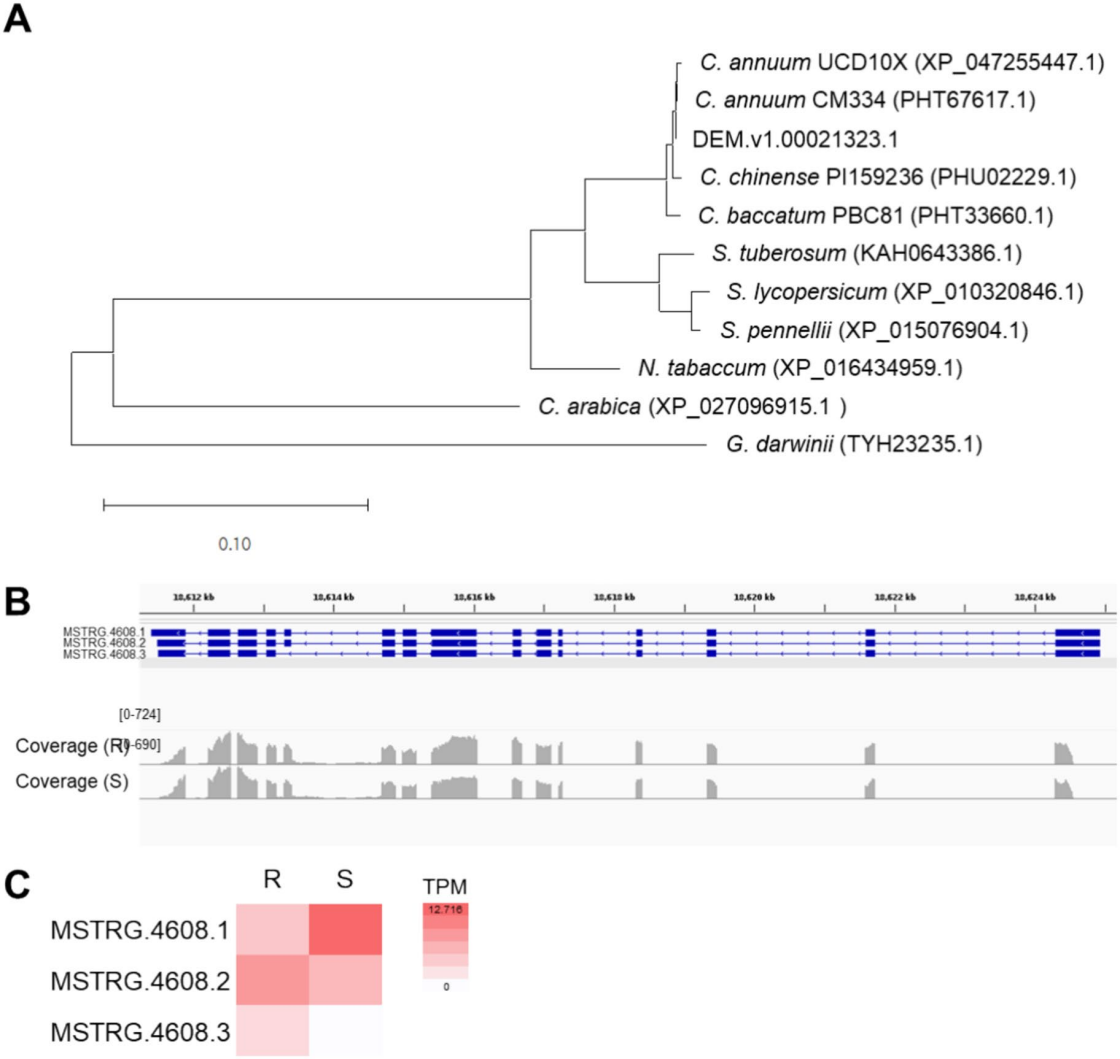
Sequence and transcript diversity of the *cvr4* candidate gene. **A** Phylogenetic tree of the *cvr4* candidate protein NOP14 encoded by DEM.v1.00021323 and related proteins from other plant species. **B** Integrative Genome Viewer browser window showing the three transcript isoforms (MSTRG.4608.1, MSTRG.4608.2, and MSTRG.4608.3 [DEM.v1.00021323]) and the coverage by RNA-seq reads from the resistant (R) and susceptible (S) pools. **C** Heatmap representation of expression levels, expressed as transcripts per million (TPM) values, for each of the three transcript isoforms of the *cvr4* candidate gene DEM.v1.00021323. White indicates low TPM values, and red indicates high TPM values

We also examined sequence polymorphisms in the *cvr4* candidate gene (DEM.v1.00021323) between the resistant (CV9) and susceptible (Jeju) accessions used as parental accessions for the original segregating population. We detected no polymorphism between the exon sequences of this gene in CV9 and Jeju (Data S2). A bioinformatic analysis revealed that DEM.v1.00021323 encodes NUCLEOLAR PROTEIN 14 (NOP14). We reconstructed a phylogenetic tree for pepper NOP14 and related proteins by querying publicly available genomes from other *Capsicum* species and Solanaceae (Fig. 5A**; **Figure S5). The phylogenetic tree indicated a high degree of conservation among NOP14 homologs in *C. annuum* and other *Capsicum* species.

A closer look at the BSR-seq data indicated that DEM.v1.00021323 produces three possible transcript isoforms (MSTRG.4608.1, MSTRG.4608.2, and MSTRG.4608.3) (Fig. 5B). Subsequent analysis of the BSR-seq data revealed that all three transcript isoforms are represented in the assembled transcripts derived from CV9 (the score tab of 1,000 in the gtf file). By contrast, we detected only two transcript isoforms among the transcripts derived from Jeju, with the third isoform (MSTRG.4608.3) missing (the score tab of MSTRG.4608.3 from Jeju in the gtf file was “.”) (Data S3). From these assembled transcript datasets, we calculated the TPM values for each of the three isoforms using stringtie2 (Fig. 5C). The three DEM.v1.00012323 transcript isoforms were expressed at variable levels between the resistant and susceptible pools. For instance, MSTRG.4608.2 is the most expressed transcript isoform in CV9, whereas MSTRG.4608.1 is the most highly expressed transcript isoform in Jeju.

## Discussion

Here, we characterized the ChiVMV-resistant pepper cultivar CV9 carrying the new ChiVMV-resistant locus *cvr4*. We identified and fine-mapped the *cvr4* locus in CV9 through BSR-seq analysis. Among the *cvr4* candidate genes, DEM.v1.00021323 encoding the nucleolar protein NOP14 emerged as a possible candidate for the *cvr4* gene, as its VIGS-mediated silencing in the potyvirus-susceptible cultivar Jeju resulted in increased viral resistance to ChiVMV. Although we did not identify any SNPs in the coding region of this gene, the BSR-seq data showed evidence that this gene is expressed as multiple transcript isoforms with different expression patterns between the resistant and susceptible pools, which may relate to the ChiVMV resistance seen in CV9. However, because BSR-seq could only observe the major QTL region in the CV9 × Jeju population, the other approaches, such as QTL mapping or BSR-seq with independent populations, to detect the minor QTL for ChiVMV resistance.

Potyviruses represent one of the most significant threats to global crop production (Yang et al. 2021). Due to the mixed infection problems associated with potyviruses, it is imperative to develop breeding materials with resistance to potyviruses (Barbosa et al. 2016; Goldbach et al. 2003; Gray and Power 2018). CV9 represents one such promising genetic resource for breeding pepper varieties that are resistant to potyviruses. Our characterization of CV9 revealed that it exhibits broad-spectrum resistance to the potyviruses ChiVMV, TEV-HAT, and PepMoV-NIb. The above information could be helpful for resistance breeding in pepper.

We propose that the *cvr4* gene encodes the nucleolar protein NOP14 based on our mapping data and supported by our VIGS analysis (Fig. 4). NOP14 is responsible for nucleolar processing and export of precursors of ribosomal RNAs to the cytoplasm (Dragon et al. 2002; Milkereit et al. 2003; Sáez-Vásquez and Delseny 2019). Many plant and animal viruses hijack the host cell machinery for their survival. The nucleolus is an essential cellular component during virus infection (Hiscox 2002, 2007). Indeed, TEV P1 protease accumulates in the nucleolus during the early infection stage and physically interacts with 60S ribosome proteins during infection (Martínez and Daròs 2014). Similarly, the CP P1 of cucumber mosaic virus accumulates in the nucleus and the periphery of the nucleoli in infected cells (Lin et al. 1996). The plant pathogen Groundnut rosette virus interacts with the fibrillarin in the nucleolus via its open reading frame 3 (ORF3) for its systemic infection (Kim et al. 2007a, b). These findings underscore how ribosome processing in the nucleolus might play a pivotal role in plant virus infection and NOP14 may directly or indirectly interact with ChiVMV proteins.

Transcriptome assembly and quantification revealed the existence of three transcript isoforms for *NOP14* and their distinctive abundance between the two resistant and susceptible cultivars (Fig. 5). Splicing variants from alternative splicing (AS) are one of the many eukaryotic strategies to broaden the complexity and diversity of their proteome (Hewezi 2024). Plants require efficient and timely responses when facing abiotic or biotic stresses for dynamic transcriptional and post-transcriptional reprogramming of gene expression. Global transcriptome profiling studies have demonstrated that AS is actively involved during biotic stresses in many crops (Gervasi et al. 2018; Gui et al. 2022; Laskar et al. 2023; Zhang et al. 2019). The expression pattern of splice variants is markedly different between resistant and susceptible apple (*Malus domestica*) cultivars against *Alternaria alternata* infection at 36 and 72 hpi (Zhou et al. 2022). In plant virus resistance, only the *N* gene is known to be expressed as two splicing isoforms, *N*_*S*_ and *N*_*L*_. Both isoforms are necessary for complete resistance to TMV in tobacco (Dinesh-Kumar and Baker 2000). In the DEM.v1.00021323 gene, three transcript isoforms were identified (Fig. 5B). Among the transcript isoforms, MSTRG.4608.3 was only found in the resistant bulk pool, which might be involved in the ChiVMV-resistant responses (Fig. 5C). Instead, the expression level of the other transcript isoform (MSTRG4608.1) was slightly higher in the susceptible bulk pool. These findings support the idea that variable expression patterns of the *cvr4* transcript isoforms may be related to biotic stress responses.

The *cvr4* allele in CV9 is recessive and confers resistance to ChiVMV infection (Table 1). Most recessive resistance genes are host factors for successful viral infection. For example, because eIFs interact with potyviral VPg proteins (Kang et al. 2005b; Sanfaçon 2015; Zhou et al. 2024), gain of resistance (or loss of susceptibility) is acquired by their mutation (Rollwage et al. 2024). For this reason, these genes have been termed *susceptibility* (*S*) genes in crops. Many biochemical approaches have identified the S proteins that interact with potyviral proteins (Jiang et al. 2015; Vijayapalani et al. 2012), but only a few *S* gene candidates have been genetically characterized in potyvirus infection (Castello et al. 2010; Ouibrahim et al. 2014). Here, we identified the *cvr4* gene as a previously uncharacterized *S* gene candidate against potyvirus infection.

Recent advancements in gene editing have enabled the engineering of *S* genes to develop virus-resistant crops (Garcia-Ruiz et al. 2021). For example, mutations in *eIF4E* can confer potyvirus resistance in wheat, pepper, and tomato (Kan et al. 2023; Kuroiwa et al. 2023; Yoon et al. 2020). In addition, the simultaneous targeting of three *S* genes (*Pi21*, *Bsr-d1*, and *Xa5*) resulted in enhanced resistance to rice blast and bacterial blight (Tao et al. 2021). Gene editing of *Mildew resistance locus O* (*MLO*) also conferred powdery mildew resistance without growth penalties (Li et al. 2022). These examples illustrate the potential value of engineering *S* genes for breeding virus resistance in the future and the possibility of accelerating the discovery of *S* genes against viral infection (Ruiz-Ramón et al. 2023; Shopan et al. 2017). The *cvr4* gene encodes the NOP14 protein that may be involved in ChiVMV infection. Gene silencing of *cvr4* (DEM.v1.00021323) produced complete ChiVMV resistance in a susceptible cultivar, although this resistance was accompanied by an abnormal growth phenotype (Fig. 4A, B). These results suggest that the *cvr4* gene may be used as an important gene editing resource in pepper for potyvirus infection. To avoid the growth penalty, it will be necessary to judiciously edit *cvr4*.

In this study, we identified the resistance gene *cvr4* in pepper. This recessive resistance gene may be used as a potential target gene for editing to acquire resistance by its mutation. Further gene editing studies of *cvr4* may accelerate the breeding of potyvirus-resistant peppers in future breeding programs.

## Supplementary Information

Below is the link to the electronic supplementary material.

Supplementary file1 (XLSX 20 KB)

Supplementary file2 (DOCX 18 KB)

Supplementary file3 (DOCX 2469 KB)

## Data Availability

The datasets generated and analyzed during the current study are available from the corresponding author upon reasonable request.
