# Supplementary material for: Fine mapping of the Chilli veinal mottle virus resistance 4 (cvr4) gene in pepper (Capsicum annuum L.)
Source: Theor Appl Genet. 2025 Jan 7;138(1):19. doi: 10.1007/s00122-024-04805-8 (PMC11706928; doi:10.1007/s00122-024-04805-8)
Supplement: Supplementary file 2 — Supplementary file2 (DOCX 18 KB) [file 122_2024_4805_MOESM2_ESM.docx]

**Data S2. The exon and intron region of the DEM.v1.00021323 gene.** Highlighted regions are the region sequenced by Sanger sequencing but not polymorphic between ‘CV9’ and ‘Jeju’. Red characters represent the predicted exon region in the reference genome, ‘Dempsey’.

>DEM.v1.00021323_exon_with_intron

ATGGCGAAAACGAGCAAAACCGAAAGTTCATCAAAGAAGAAGAAGATGAAGAAGAGCAGCAAAACAGGTCCAAAATCCAAAGACATGAAGCTCAAACCACCAAAGGAAAATCCATTCGAAGCTATTTGGTCACGCCGTAAATTCGATATACTTGGCAAAAAACGTAAAGGTGAACAACGAAGAATTGGTGAAGCTCGTTCTTCTGCCATCGAAAAGGTACACTTACTTTTCTTATTTAGTCTGTTTCAAAGTCTGACAGTATTTAAAAGTCTTTTATTACTTTCTTAAATTTCGTGCCAAAGTGAAAACGAGACAATTAACTCGAGACAGACGGAGTATTTAGCATAAAAAAAAAGTTGCATATTTTTGATTGATTTTTCATTAATTTTAGGTAGTTTGGTAAACTACTTAGTCATAAGAACTATCTATTTTGACTCGACAAGAAATTTAAAAAAATGAAATTTTTTTGAATTTTGTGATCTTAAATTAAAGTTGTGTCAAATGTACAAAAAGCCTTTTAATCTTGTAGTTTCAAACATGCCATTTGGAAAGTTGGAATTGAAGTGTTGCCAAAAAGGAAAAAGGGTCATTCTTGTTGAAACAGACTAAAAAGAAAGTACTTTAATTTTTTTGGAATGGAGGGAGTACAACATTCGAGAGCTAGTGAGAAAATAAAAGTTATTCATATAAAAGATTAATATGAAATTTGGTTTGCAATTTAGAAACCTAGATAACTAATACTTGCATAGTTAGTAGCATTACTAATACAACAACAACAAACCCAGTATATTCCCACATAGTGGGGTCTGGGGAGGGTAGAATGTACGCAGTCCATACCACTACCTCTAGGGAGGTAGAGAGGCTAATTCCAATAGACCTCCGGCTCAAGACAAAAGACATTAAACAGAAACATCAAAATCGTAGAACATGATACAAATAAAATAGGTATGACACTCGCAATTAGAGAAAAGAAAGAAAGAAATACATACGTAAATCAATAACTAAAGCCATAAATAAATAGATAAATACATAAATTTGCCAACAAACGACAGCAAGTCCTCCTTCTTACTGACTACTACTCCTCCACACCCTTAGCCCTTTACTCTAATACTTTTCCTCCATAACTTTCTATCCAGGGTCATGTCCTCAGTCAGCTGTAACTGCTCTATGTCACGTTTAATCACTTCTCTCCAGTGTTTTTCGGTCTACCCCTACCCCACTTGAAACCTTCCAAGGCCAACCTCTCACACCTACAAACTGGGGCATCCGTGCCCTTCCTCATCACATGACCAAACCATCTCAACCTCACTTCCCGAATTTTATCCTCCACCGACACCACTCCCACCTTCTCTCGAATAATTTCATTCCTAACCCTATCAGCCCTAGTGAATCCACATATCCAGCGTAACATTCTCATTTCCGCCACCTTCAACTTTTGGATATGAGAACTCTTAACCGGCCAACACTTTGCTCCATACAACATAGCAGGCCGGACTGCAACTCTATAGAATTTGCCTTTCAGTTTGAGGGGCACCTTGTGTCGCATAAAATTCCCAAAGCGAGCCTCCATTTCATCCAACCTGCCCCCATGCGGTGAGATACATCCTCATCTATCTCTTCATTCCCCTGAATCGTAGAACCAAGATACTTAAAACTATCCCTCTTGCAAACCACCTGAGAATCCAACTTTACTACCAGCTCCTCCTCTTGCCTCGAGTCACTGAACTTGCACTCTAAGTACTCCGTCTTGGTCCTACTCAACCGAAAACCTTTAGACTCCAGGGTCTCTCTCCAAATCTCCAGCTTATCATTAACCCCTTGACGCGATTCATCAATCAGAACTATATCATTCGCGAAAAGCATACACTAAGGCACCTCACCTTGTATATTCCGCGTCAGCACATCCATCACCAAAGCGAATAAAAACGGACTAAGAGTTGATCCCTGGTGCAACCCTGTCAAGACTGGAAAATGCTCAGAATCTCCTCCCACTGTCCTTACCCGAGTCTTCGATCCCTCATACATGTCCTTAATCGCTCTGATATACGCCACGGGAACCCCCTCGCCTCCAAGCAACTCCAAAGAACCTCCTTAGGGACCTTGTCATTCGCCTTTTCCAGGTCTATAAACACCATGTGAAGATCCCTCTTCCTCTCTCTATGCTGCTCCACCAGTCTCCGCACAAGGTGAATTGCCTCAGTCGTGGAGCGACCAGGCATAAAACCAAATTGGTTCTCCGAAATAGACACAATCTTCCTCAACCTCCGCTCCACCACCCTCTCCCAAATCTTCATCGTGTGACTCAACAACTTAATACCCCGGTAGTTGTTGCAACTCTGAATGTCACCCTTGTTCTTATATAGAGGAATCATCGTGCTCCATCTCCACGCCTCGGGCATCTTTGCTGACTTGAAAATATTATTAAACAAGTTGGTCAGCTACCGCAACCCTGACCCGCCATAGAACTTCCAAAAATCCACCGGAATCTTGTCGGGCCCCGTCGCCCTACCCCTTCTCATCTTGCGAATAGCCTCACTGACCTCCTCTACCCTAAAACGCCGACAGTTAGTAGCATTACTAATACCTGCATAATTCTAACCAGCTACCAAACGACCTTAATAATATCTATAAAAGATGTTGTTTGCTTTCTTAATTCTGGGAGAAGCCAAAACCGTATAATCAAATTGAAAGAGTGAGTATTTAAAAAAAAAAGTTGCAGTATTTTGACTGATTTTCCATTAATTTAAGGCTTTTGGGATTTTGATTATGATTGTTTTGTTGTGATTATAGAGGAAGAAGACACTATTGAAGGAGTATGAGCAAAGTGGGAAATCATCAATGTTTGTTGATAAGCGTATTGGAGAAAATGATGAAGGGCTTGGTGAATTTGATAAAGCTATTATGAGGTCTCAGCGTGAGAGGCAGGTTTGTTTCGAAAGTTTTCTTCACTGCCAATCGTTTAATTTTGTTCATATTTTGTTGTAAGAAGTAAAAGTTGATGATGCTTCATAACTCACAATCAATGTTAGCATAAAGTAGTGATTTTTATCATGTTAGCGTGAAGTAGTGATTTTTAGATGATAATGTCTTCTCTAAAAGTTCTGTTCACGACCAATGCTTGAATATTATATTCAGATTCTACTTTTTTTTTTGAGTAAGCATGAGCACCTCGACTAATTCAATGAGAAAGCTCAACTAGTTCCATTATAGGGGTACTTGCTAAATCTCTCACCATACCATTACATGTATCGGTGTACTCTGTCTATCAAGATTTGGATGGATGAGAAGGATTCACCTAGTTTTTTGCCTCTCTGCTGGAATTTGAACATGAAACTAATGGTTCTAAATCCACTTCGTTTATGAGTAGGTCTCACCTTGGGTGTCAAATTCTATAGTTAGAAGTAACATTTGATGTCCAAAGTTGTAACCTAATGGTTGATGTAATGACCCTTTCGGTCATTTTTCATATTTCCGCTTATTTTCACCGTTAGGGCCTTTCCATAGCTGCCACAAGTCAAATGACTTGTGGGATGACTTGCCTTTCCATAAAAATGACTTGTGACAGCTATGGAGAAGCCCTAACGGCGAAGATAAGCGGAAATATGAAAAATGACCGGAAGAGTCATTACAGTTGATGAAGTGGGAGGAGAACTATGTGGCTTCAAGTTTAAATCCCACACTATTTTTTTTTTAAGGTGTTCTTTTCCATCCACCTTAGTTTAGGTGGCCAGTTACTCGGTACGTGGGAGGTACAGCCTGGTGGAACAGTTGAGAGGTCTGTACACCATTGTCAATAAAAAAAGAGTTAAAACTGATGCTTCAGAAGTTCAACGTCCTCTTTTTGTATTGCCCCCACGTGTTTGATGGGGAAAAACTTGTTCTCTGTTCGTTATGTTAGCATAAAGTAGTATTTTTTATGGTGGAAAATATACACACTCATTGTAAATAAAAAAGAATCATAAGTAGATGTTGAAGAAGTGGTCTTTCAATGGCAATCATGGAGATTTAGTTAGCTATGCTTTTTTAGTTTGGCCATCTAAATAATGAAGAGTCAGTTAGAGAAGATTAGAGCATTGGCGTGAGAGTGAATTTTCGGTCAAAATAAACAAGCTTGTTAAATGCCAAAATTGAAAATTGCACGTCTGGATATTTGAATACGCCAACTTACATAATGGAACGGAGAAGGCTCAACCCTCCATAGTGAACCCAACTTTGGAAATGGGATCTGAAACAAATCAATGAACTCATTTTCAAACAAAGAAGATAAAAAAGAAAAGCGTGGAGCCTAGGAATAGGAAATGAATGGATCATTGCGTGTTCATTTCGTCAGGAACGCCAGATTAATCATCATTAGAAAAAAAATGCGCATTGAGTGAGTAGTTAGTTGAAGGCGAGCCTTGACTGGTAAATTTACTGCCATGTGACCAGGAGGTCATGTGTTCAAGCCTTGGAAACAGCCTCCGCAGAAATGCAAGGTAAGGCTGCGTACAATATACCCTTGTGGTGAGGCCCTTCCTTGGACTCCGTGCATAGCGGGAGCTTTAGTGCACCGGACTGCCTTTTTTTATTGTGCGAGTAGTTAGTTCAGGCCTTTTTGCATGAGAAAAATGGTTGCATGATTTTACCAGTGTGCCTTTCACTTTTATTAAATTATGTTCATTTCGTCAGGAACGCCAGATTAATCATCATTAGAAAAAAAATGCGCATTGAGTGAGTAGTTAGTTGAAGGCGAGCCTTGACTGGTAAATTTACTGCCATGTGACCAGGAGGTCATGTGTTCAAGCCTTGGAAACAACCTCCGCAGAAATGCAAGGTAAGGCTGCGTACAATATACCCTTGTGGTGAGGCCCTTCCTTGGACTCCGTGCATAGCGGGAGCTTTAGTGCACCGGACTGCCCTTTTTTATTGTGCGAGTAGTTAGTTCATTTGCATGAGAAAAATGGTTGCATGATTTTACCAGTGTGCCTTTCACTTTTATTAAATTATCTGATACTGCTCTATTGTTGTTGACAAATATCTACTTTTTGTTTTCCTTAATATCTAGATGAAATTGAAGAAAAACAAGTATAATCTGTCAGATGAGGATGAAGAGGACTTTGACTTTGGTGTCTCATTGGGAAGGGATGATTTTGATGAGGAAGTTCCATTTGATGAGGATGAGGAGGATTATGCAAGAGACGGTGAGAATAACCCTTCGCGTGTTTAAAATGATACTAGTTTTTATATGGCAAAAAAAGGAGTAATTTGTTGCCAAAATGTCATGTTGAATTCAAATCATACTAATTAGGATTCTTTGAAATTATAGAGGGAGAAAATCTTTTTGTTCGAGAATGTGTGCGTAGCTTATTGGGGTAGACCAAGAAAGTTGGTCAATGTCATTGTTTCAATGGAGTACTTTTTCACTTCTTGAATCAGGGTAGGAGAGACGTATGGTATTATTTTTAGTTTGGTTCCGTATCCTTTATGGTAAGGACTAAGGACACCTATTTTATTTTATTGTTAGATGCCCCACCTTTTTTGGTAGTTACGCATTAACATTCTTAAGTCAATGTACATTTAAAAGAAACCACAGAGCTTTTGATGGGGTGGAGAACTACTATGTACATTTGAAGAATAGTCTCTCATTCCCCGTTTCTTTTGCTGCATCAGTGAAGTTCCAGTTTGTATATAGGAGCTGGTATGTTCAGATTTTGTAGATTCTCTAATTTTCTGTATACTGTTTGTTTGCTGGAGATTTTCGTATGATAGTAAATTATTTACCTTATTAAAAAAAAACCTCAAAGTCCCTTGTATAGTTACAAAAGTTAAGGAGAATGCTTCTGATGGTCCTGACACTCTTGGAGGCTTGTACTATCTTTTTGCTGTATTTTGTCGTACAAGTTACTCCCTATTCAAAAAATAAATAATAGGAGAAAAATTTATGAAGAAAAACCATCATCATAAACCTAATCTAGCTACCAGCTAGAGTATTACCACCATTATTGGTTCATACATTATCATATCTTTTTTTCAGGGAAGTTGGTAATGTTTCAAAGCTTGTATTTTCTGTTGTTTAGCATCTTAAATTGTTTCTTGTATGGTTTTCCTCATATGTCTATTTTGGTGCAGATAAGTCAGCTATCTTGGGACAACTTAACTCTCATGGATCTCAGAATGCCCAACCAGGTCCAGTGGAAGCAGAGGAAAATGTTAGAATCTCCCCCTCCCCCTTTTCTTCATGCATATTACATGCTCTATTTTGGAGTTTATGGTGACTAGCTATTAACAGAGGAAATAGTTGTTATCTTTCTTGCCCCTCCCATGATTAAGAGCTCACTTAATTGTTATTCTGAATAGTTTGGCACTTAGATATAAATGTTTGTAACCTTGTAACTTAAATTGCTACTCCATGCATCTTTTGAGTCATATGAAAAGAAAGAAATTGAACATGTCTTGAAGGAAGCACTGAATCTACACACACGATGAAATATAGTGTCTCTATCTTTTCGCTTTTTGATGTTACCTTATAAAAAAAAAACCTTTTTGCTTTTTGGTGTAAGTTGATGCATTACTAGATGTACACATTATTGATGCATTACTAGATCATTGCAGTACCCTACAATTGTAAAAGGTATATAGGGACTGAGATATTCTTTTTGGCTGTACAGAGAAGCACCAACTTGGTGCAATTTCATCAATAAAATTATTTACTTGTGAAAAAAAATTGTTTTGCTCAAGCAAAGCTGTTAGGTGGCCGTGTTAATTTTTGACTGATGATACTAAACTAGCTTATGCCTCTGATAAAATGTCATTTAAACATTGTTAACAAGATAATTACATATGGGACCACTGATTTTACAGGCTGCCATTTAAAACAACAACAATAACAACAACAAACCCAGTGTATTCCCACATAGTGAGGTCTGGGGAGGGTAAGATGTACGCAGTCCATACCTCTACCTCTAAAGAAGTAGAAAGGCTGTTTCCGATAGACCCCCGGCTCAAGACACGGAATACTACACAAATACATAGTAAAGCATGGAACAGGCTGCCATTTAAAACCTGAGTAATTTATGAAAGAGAGAAATAAGAATAAAGATTTAACAGAAGAAAACAGGTTCTTACTTTGTTACCGGGAGCATGCATCATCTTGCGTGGGATAATAGTCCATTGTTCACAAACATGTCTCGAAAGATTCCTTTTTTTTTATTTACAACACAGCTGTTGTTCCACTCCTGATTTAGTGTACCATCTTATTGCAGTGGAAGAAAAGCAAGAAAGAAGTGATGGAGGAAATCATTCAAAAGAGTAAATTCTTTAAGGTTTGTTGTTGTTTAATGTCTTATGGTGCAATCTGGTAAGCGAATGTCTCTTTACTTGCCATATGTTTGACAATTCTTTCGTTTTCCCTACATTGTTAGGCCCAAAAAGCGAAGGACAGAGAAGAAAATGATGAATTAACTGAACAATTGGACAAAGACTTCACATCACTGGTGAATTCAAAAGCCTTATTGTCACTGACCCAACCAGACAAGATAAATGCTCTGAAGGCTCTTGTGAACAAGAACATTTCAGTTGATAATGTCAAGAAAGATGAGGTAGCTGATGCTCCCAGAAAAGGATCTATTGGAAAGGTATAATTGGGTTTAATTTTTGATTGCCAGAAACATTCCAGATGGATATGCAGAATCCTAGTCTTCCCTCTTTTCGTGTGCCTCAATGTATCTTCTTCTCCATCTCTTTTAGGTTTCTAGTGCATTGTCCATGAACAATTACCAATAAATTTTCTTTTGATTATATTAAATTTATCTATGTTGACCACTTTTATCTTCGTATTGCTTTCAGGAAAAGCCCGACACTTATGAGATGCTTGTTAGTGAGATGGCAATGGATATCCGTGCTCGCCCATCAAACCGGACAAAGACTCCTGAAGAAATTGTGCAGGAGGAGAAAGAACGGCTGGAATTATTGGAGGTACAGATGCATTATGGCAGAAACAAGAGCTCTTGTGTCTCTTTTTAGCAATCATGCATTACAATGAGAATCTTTGGTACCAAGATGGAGAAAATGGTTATATTTCTATTAGTAAATTGCACTATCAAATTCTAAACTAAAGTCCTTTAAAAAAATGTTTTCTTTTTGACGATAGAGGATCCTGGTTCTATTTGACTATTTATAACATTCCTTCTGTGATGGTATCCTTATGTTCTTTCAAGTTGTTGTAATGTATGACTTTGTTATCAGCTAGGTAATTGGCGTTCTTGCATCATCATTACTGTTCTTTCATGCCTTCATTATGAAGCTGCCTACTGTGTAGTAGTAGCGATTGCTTGCTTTGTGCATTTTCACATTGTAGTCTTCTAAAACAGAATACATTTAGCTGAACTATTGGTTCAAAGATTTAATGGTTTGGAAGGATAAAGAACATTTATCTTTTGGCTATAGTAGATATTTGCTAATCAAAGTTTTATTCATTTTGCAGAAAGAACGTCAGAAGAGAATGGCTGCAGTTGATGATGAGAGTGATGAAGATGGAAATGCATCTGATGATGACAGTAAATTGGTCAGAGACCCAAGGACTATATCTGGCGATGATCTTGGGGATGATCTTGAGGAGGCGCCTAGAAATAAGTTAGGTTGGATTGGTGAAATCTTGAGAAGGAAAGAAAGTGAACTTGAGAGTGAAGATGCTGCTTCTACGGGGGATTCAGATAGTGAGGAGGACGATGGTGAGGATGAAGGAAGTGACGATGGGGAAGAAGAAGGAAGTGGCAATGGGGATGATGAAGGAAGTGATGAGTATGAAGAAGGAAAGACACAGACTATTAAGGACTGGGAGCAAAGTGACGATGATATTATTGATGATGCGAAGAAGGTGATGAAAATAAAGGACCTTAAGGGGGTTGACGTCAAGGGAAAAGAAGTTGGCACTGTACAAACCAAAAAAGAAAAAACGACTTTGAAGCATCCACAAAGCGAACTTCCCTATACAATTGAAGCCCCCAAAACCCTAGAGGAGTTCACTTCATTGATAGACAATTGTTCTGATGATCAAGTAATTGAAGCAATCAGGAGAATTCGTACATTCAATGCAATAACTGTTGCAGCAGAGAATAAAAAGAAAATGCAAGTATGATACTCTGTTTTCACTGCAATTTCTTTATGATTGATGTTTTAACCTTTGAGACTTTCTCCGTTCTTGTTGTTAACACATGGTGTGAAGGTTTGCCTTGAAATTTTAAGAGTATTTCACCATTTAACTGTATTTGCTCATGTAAATATCTCTGTGCATTGATAAAAAATGTTTACCGCAATTTCTTTATAAGTAGCTTGTTTCTTTCATCAGGTCTTTTACGGCGTGTTGCTGCAATATTTCGCTGTGTTGGCAAATAAGAAGCCATTGAATTTCAAGCTTTTGAATTTGCTTGTAAAGCCACTGATGGAGATGAGTGCAGCAACCCCATACTTTGCAGCTATATGTGCTCGCCAAAGGCTGCAAAGAATAAGGACACAGTTTTGTGAAGATCTTAAGCATACAGGTTTGTTTTCATATCCCATTTGTTTTCTTTAGTCAATCATTCTATCTCAACTTATGTTTCTTGACTTCACCTATTGTGTTTAATTCATTTGGTCCATCCGCATAGGAAAAAGCAGTTGGCCATCTTTGAAGACTGTCTTTTTGTTGAGGCTTTGGTCCATGATATTTCCATGCTCTGATTTTCGCCATTGTGTCATGACTCCTGCTATACTGCTTATGTGTGAATACCTAATGCGCTGTCCCATCATTTGCGGAAGAGATATTGCAATTGCTTCCTTCTTGTGCTCTTTGCTGCTCAGTGTAAGTCGATACCATGTTTCATTTGTCTTTCATGCACTGAAATTGATCGTTTGCTTAAGTTGAGGGTTCTACGATTTTGTTTAGTAGTGTCTTATATATCATCGGAACACGTATGGTTTCCACCTGTATTATAATCTTCAACTAAATCAAAGCAGCATCTGGACTTCTGGATGTATAGTGATTGTCTCCGCCTAGTATATATCGTGTACCTTACTAATAGTATGTGCTTCATAGTGAATATCTGCATATTACCCTCCTTGTTCTAATTCGTCAAGTCATTTGTCTTTGAAACCATTTATAGATATCTCCATTGTTTCTCCTTCTTCCTTCCTTGTTTTATACTTAAATACAGCGTGTTAGTCCAGCGTTATACAGGATTTCTTAGAGAGCTAATTGCTTTTAAATCATGACTTTGATTGCAAATATTTTCAATATCTTGTGCATATTTGCTTATATACCTCTTATTAGTTTTCATTTGTGATATTTGGAAGTATGAATTCTTGTGTGGTGTGTTCTGTCCTCATACTCTCGAGGCATGAGTTCATAGCTTTGCTTGTTTTAGGTATACAAAGCTTCAGAAACTTTGTTAATTTTCTAAGATTCCATCTGTCTCTACTTATTTATTACTCTTTTGTCTGTTCCGGATTGCACCTTTTTATTTTTCAATAGCATATAGTTAAGTTTACATTCTCATCTTGTCATTTGACAAGGTTTTGTTGCAGTCTCCACTGGAAATGAAAATAAATAATTTAACTTGGTACTGAAGTAAATTTTTATATTTTATTCCTATTAAATTATTTAAATTGGAATAGAGGGAGTTTCTTTACTAAAGTCACTCAAAATCAAGGTGCAGGTAGTTCTTAGGACTTCTTTAGGTTAAATGGACCTAGATTATTTGCAATAGTCGACTTGTCATCCCCTTTTATACCTATGAGCTAAGTTGCTCGGACTCTCCAAAAATGTTGCTGAACCCTGTCGGATCCTTCAAAAATACACTATTTTGGGAGGATCTGCACGCACTCATTGACATTTTTGAAGAGTCCGAGCAACATAGCCTATGAGTGACCATACTCAAGTTGGTTCCGGTAGCTATGTGTTATTTAGTGTCAACTTCTGGTCAAACAACACGTTTTATTAAAGACCGGTTGACAAGGTAATCCCTTTTGAGATTTAACTTTTGGCTTTTAGTTCTTTTATTCTCAAAAAGCATTCTTCCTGGTTGATGGTGTTTGCAATTACAGAATGACCATTTGGACGTATTTCTCCAAGCTTAAACGTCTTGCGCTGCAGGTAACTAAACAATCTCAAAAGTTTTGTCCTGAGGCTATAGTATTTCTTCAAACTTTGCTGATGGCAGCTCTAGACAGGGAACAGCGATTTGAAAATTTACAGGTTTGGCTTTACTTCAGTGTTTGAGCCGAGGGTCTATTGGAAACAGCCTCTCTACCTCTATATTCGGGCTGCTTCCTTCCACTTGTAAATTGAAAGTCATATTCTCGTGCTGCAGTTGAATAACCTTATGGAGATCAAAGAACTTGGGCCTTTGCTTTGTATACGCAGTAGCAATGCGGAGATGGGTTCTTTAGATTTTCTTGAGCTAATGGACTTACCAGAGGACTCTCAGTATTTCGACTCTGATAATTACAGGTCAGTTGGCATTCTGGTGACATAGGCAAATCTATTTGAGGAATCTCAAAGTTCGTTAAATTTTTGAATATGGCTCTATGAGATACAGTTGAGACAAATTACATGATGCGATTAAATGTTTTCTCGTGCAGGGCTAGCATCCTCGTAACAGTTCTGGAAACCCTGCAAGGATTTGTCAATGTATATAAGGACCTTATTTCTTTTCCTGAGATTTTCATGCCAATTTCAAAATTATTATGCAAATTGTCCGGAGAAAATCATATTCCAGGTGCATTAAGGGAGAAAATCGAGGATGTATCCCAATTAATTGACACAAAGGCTCAGGAACATCACATGTTGCGTCAACCTTTGAAAATGCGTAAGAAAAAACCTGTGCCCATCAGGATGCTTAATCCGAAATTCGAGGAGAAGTAAGAGATAATCTTCTTATTGCCTTATCTTTAATCAAAAGAATCATATTGCTCGTTTCTGTCGAGCTTTACTCATCTAGTGATCATATACCTCTGCAGCTATGTCAAGGGTAGAGATTATGATCCAGATCGTGAGCGTGCTGAAAAGAAAAAGTTGAAGAAACGTATAAAAGAGGAAGCTAAAGGTGCTGCACGAGAACTGCGTAAGGACAAAGATTTCTTGGCCAAGGCGAAGGAAAGGGAAAAGGCCCTACTGGCCGAAGAGAAGGCTGAGAAATATGGGAAAGCTCTTGCTTTTCTTCAAGAACAGGAGCATGCATTTAAATCAGGGCAATTGGGAAAGGGCCGGAAGAGAAGAAGATGA
