# Supplementary material for: Fine mapping of the Chilli veinal mottle virus resistance 4 (cvr4) gene in pepper (Capsicum annuum L.)
Source: Theor Appl Genet. 2025 Jan 7;138(1):19. doi: 10.1007/s00122-024-04805-8 (PMC11706928; doi:10.1007/s00122-024-04805-8)
Supplement: Supplementary file 3 — Supplementary file3 (DOCX 2469 KB) [file 122_2024_4805_MOESM3_ESM.docx]

**Supplementary files**

**Supplementary figures**


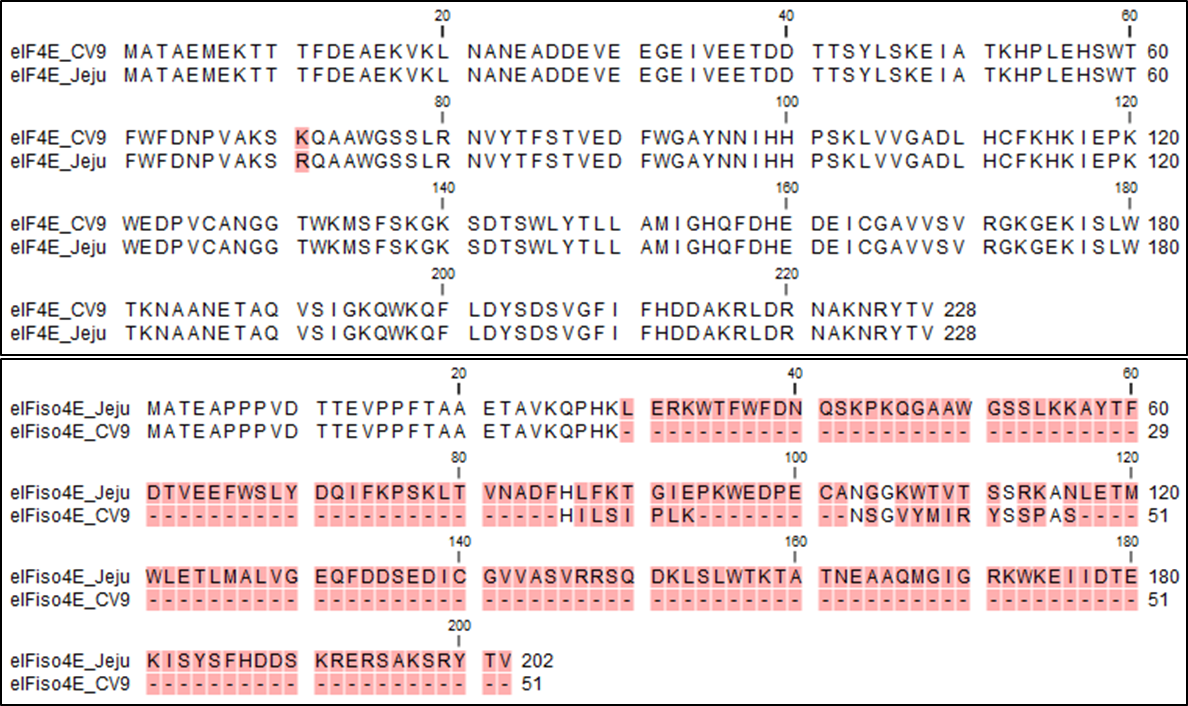


**Figure S1. Amino acid sequence alignments of eIF4E and eIF(iso)4E in CV9 and Jeju.** eIF4E is the protein encoded by *Pvr1*, and eIF(iso)4E is the protein encoded by *Pvr6* .

**
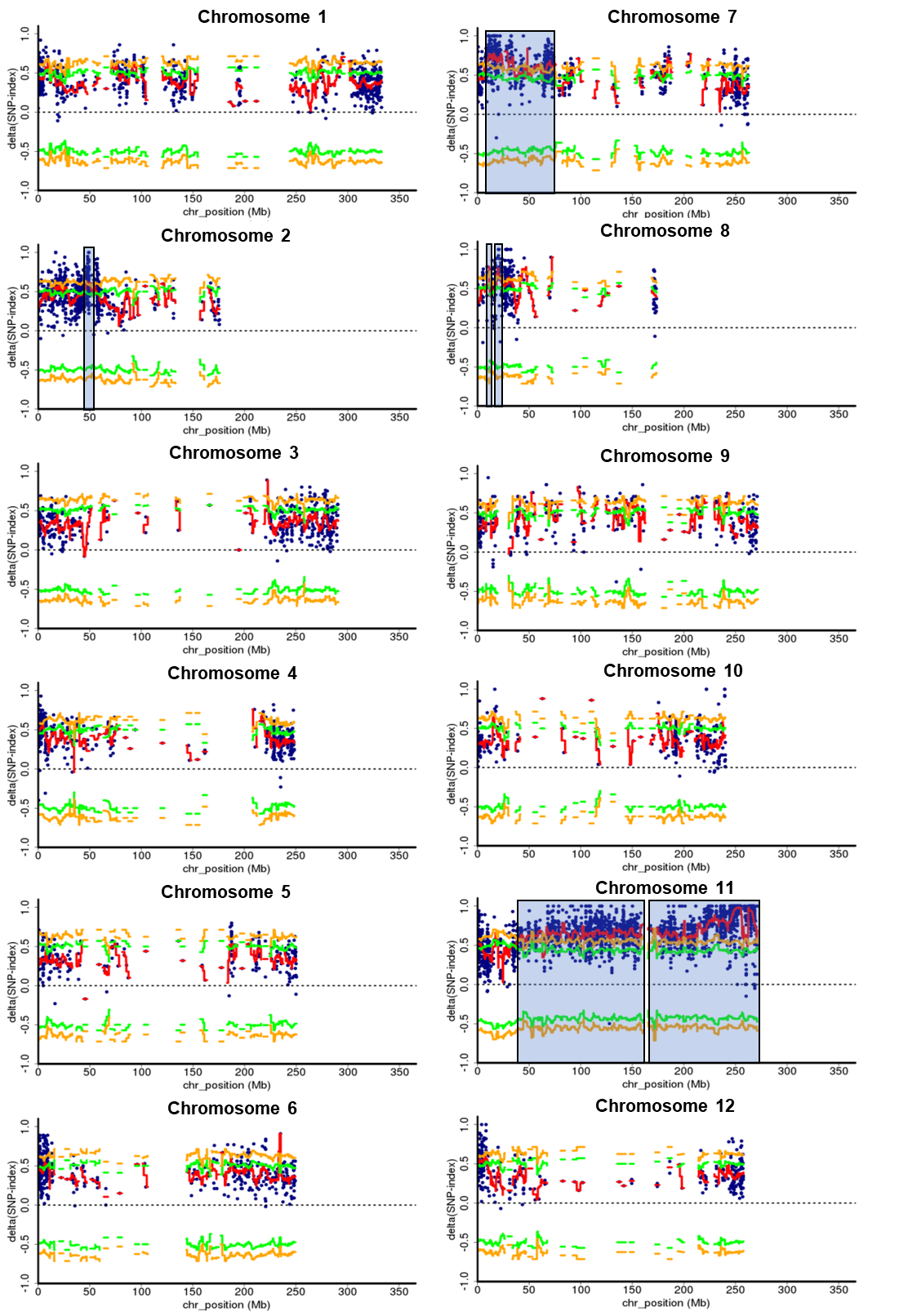
**

**Figure S2. Δ(SNP-index) plot of BSR-seq results.** The red line indicates the average Δ(SNP-index) within each window. The blue circles represent individual Δ(SNP-index) values at each chromosomal position. The green and orange lines indicate the 95% and 99% confidence intervals for Δ(SNP-index). The high Δ(SNP-index) region was highlighted by blue box.


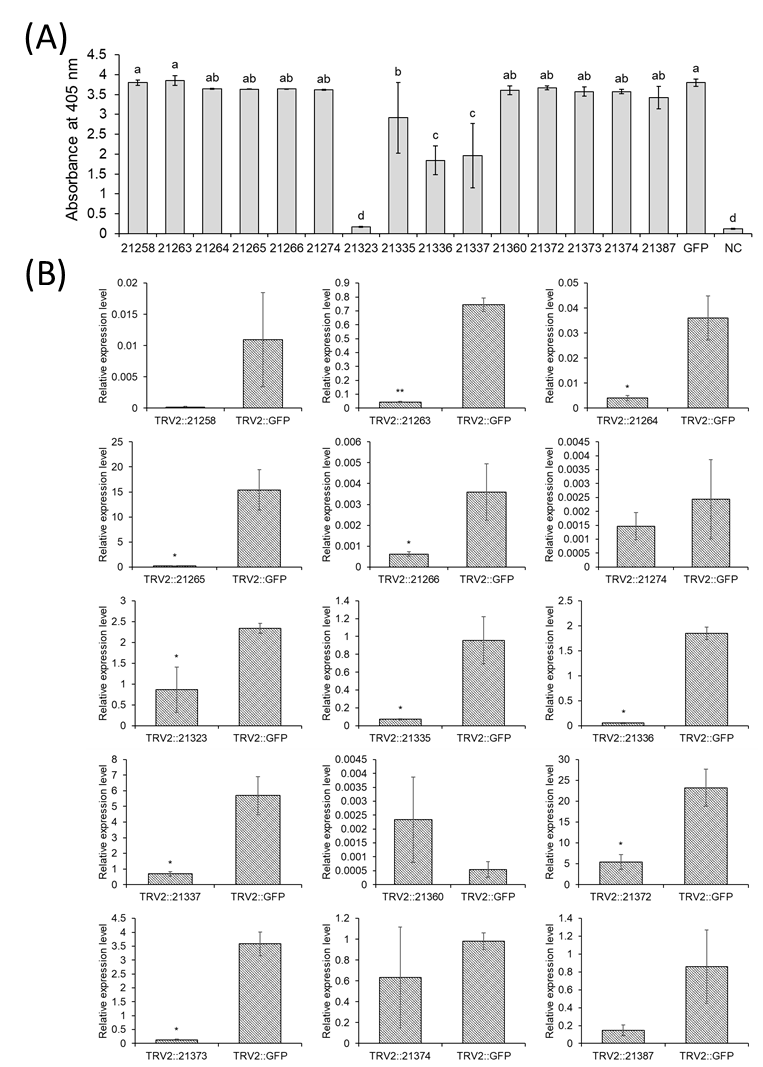


**Figure S3.** **Analysis of silencing efficiency by** **VIGS of the *cvr4* candidate genes in the leaves of the ChiVMV-susceptible accession Jeju.** (A) Viral accumulation in plants silencing for each of the 15 *cvr4* candidate genes by VIGS, as determined by ELISA for the ChiVMV coat protein. Five biological replicates were used to observe the viral accumulation levels. Different lowercase letters indicate significant differences, as determined by the Duncan’s multiple test with a *P*-value of at least 0.05. (B) Relative expression levels of each of the *cvr4* candidate genes in VIGS-silenced plants and plants inoculated with the control pTRV2::GFP. Asterisks indicate a significant difference compared to the pTRV2::GFP control plants by the Student’s t-test (^*^*P* < 0.05; ^**^ *P* < 0.01).


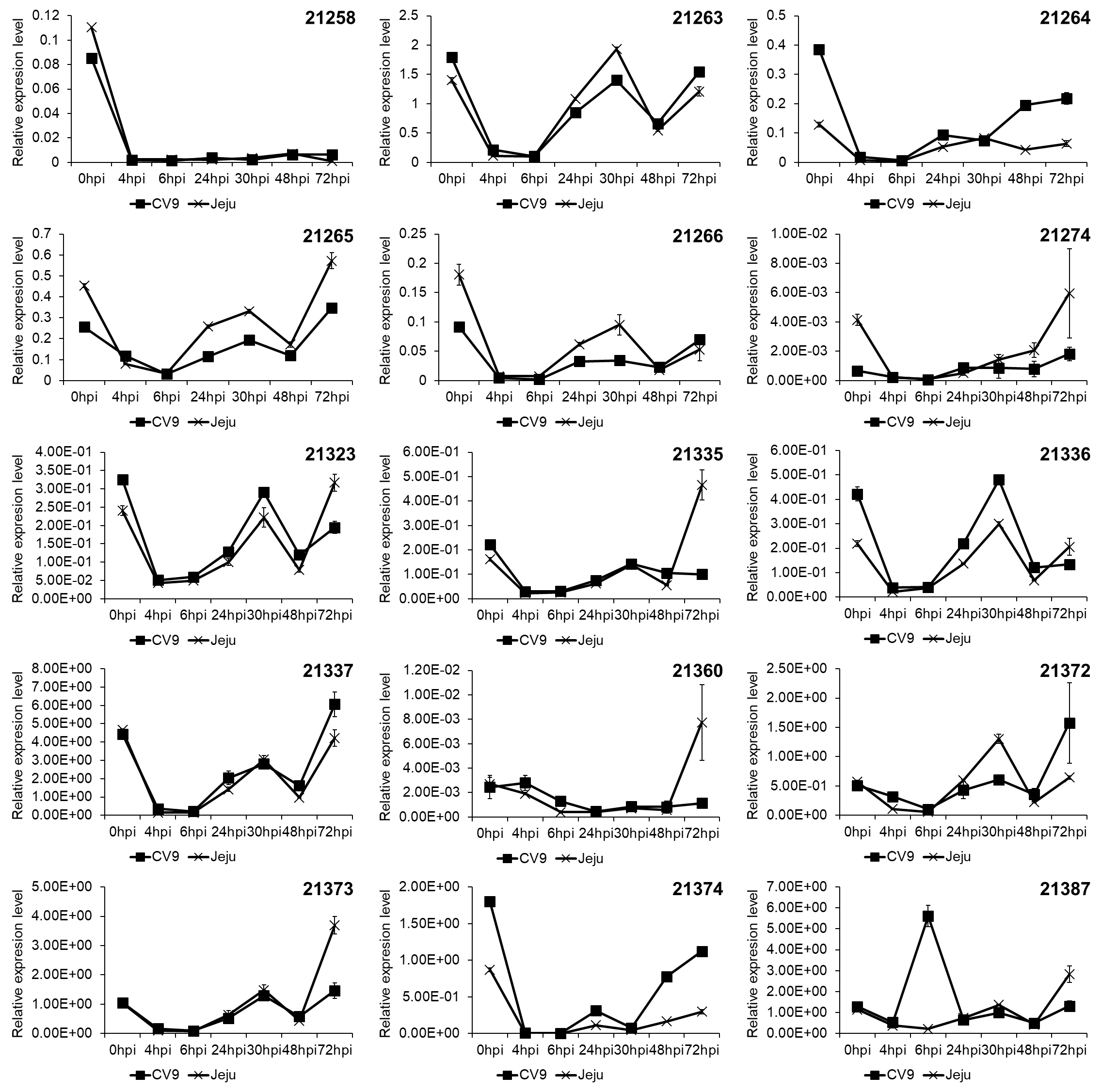


**Figure S4. Relative expression levels of the *cvr4* candidate genes in the leaves of accessions resistant or susceptible to ChiVMV.** Leaves from the ChiVMV-resistant accession CV3 and the ChiVMV-susceptible accession Jeju were infected with ChiVMV. Relative expression levels of the indicated genes were determined by RT-qPCR over 72 h post infection (hpi). Values are means ± SEM from three biological replicates.


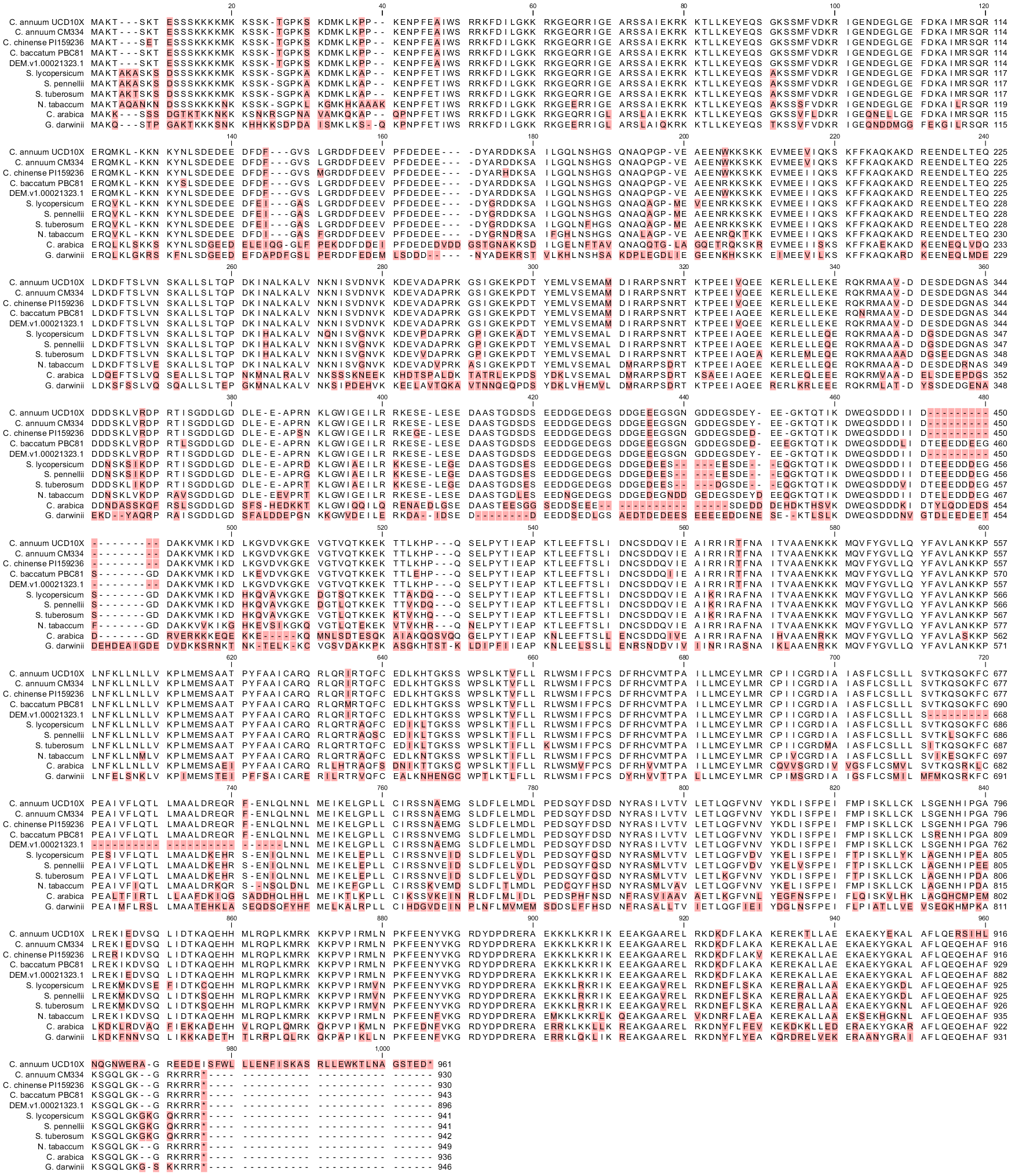


**Figure S5. Multiple sequence alignment results of NOP14 homolog proteins.** White backgrounds represent the conserved region and red boxes represent the variable peptides.

**Supplementary tables**

**Table S1. Genotyping analysis of *pvr1* and *pvr6* markers in the ‘CV9’ × ‘Jeju’ F_2:3_ segregating population**

| **Tested marker** | **Co-segregating** | **Not co-segregating** | **Correlation (%)** |
| --- | --- | --- | --- |
| *pvr1* | 42 | 62 | 40.4 |
| *pvr6* | 31 | 48 | 39.2 |

**Table S2. Summary of BSR-seq analysis of two bulk samples**

| **Information** | **R bulk** | **S bulk** |
| --- | --- | --- |
| No. of individuals in the pool | 17 | 17 |
| No. of raw reads | 193,344,588 | 161,015,867 |
| Total sequence produced (Gb) | 39.0 | 32.5 |
| Genome coverage | ~996x | ~830x |
| Number of uniquely mapped reads against 'Dempsey' | 175,097,077 | 134,946,286 |
| Uniquely mapped reads against 'Dempsey' | 90.6% | 83.8% |

R, resistant; S, susceptible.

**Table S3. Number of SNPs identified from BSR-seq analysis using the Dempsey genome as a reference**

| **Chr** | **Pre-filtered SNPs** | **Filtered SNPs** | **No. of windows** |
| --- | --- | --- | --- |
| 1 | 2,154 | 628 | 4,698 |
| 2 | 2,126 | 689 | 2,815 |
| 3 | 1,763 | 409 | 3,405 |
| 4 | 1,326 | 439 | 2,872 |
| 5 | 1,027 | 231 | 3,044 |
| 6 | 1,630 | 457 | 3,351 |
| 7 | 2,287 | 825 | 3,697 |
| 8 | 1,128 | 300 | 1,800 |
| 9 | 1,404 | 297 | 3,862 |
| 10 | 1,227 | 275 | 3,042 |
| 11 | 4,981 | 2,822 | 5,322 |
| 12 | 1,302 | 301 | 2,820 |
| **Total** | **22,355** | **7,673** | **40,728** |

**Table S4. List of candidate genomic regions for *cvr4* as determined by BSR-seq analysis**

| **Chr** | **Region (Mb)** | **Average Δ(SNP-index)** | **Average of U99** | **Average no. of SNPs** |
| --- | --- | --- | --- | --- |
| 2 | 46.75–53.05 | 0.608 | 0.591 | 76.6 |
| 7 | 8.5–73.85 | 0.673 | 0.584 | 43.25 |
| 8 | 18.25–22.20 | 0.677 | 0.641 | 20.25 |
| 8 | 29.75–30.75 | 0.628 | 0.620 | 39.1 |
| 11 | 39.4–157.65 | 0.641 | 0.552 | 41.2 |
| 11 | 175.45–270.95 | 0.736 | 0.543 | 68.1 |

U99: Sliding window average of 99% confidence interval upper side.

**Table S5. List of SNPs located within the *cvr4* candidate region**

| **Chr.** | **Pos. (bp)** | **Ref.** | **Alt.** | **SNP Quality** | **R** | **S** | **GENE_ID** |
| --- | --- | --- | --- | --- | --- | --- | --- |
| Scaffold_8;HRSCAF_313 | 252,185,010 | C | T | 27,285.38 | C/C | T/T | DEM.v1.00021387.1 |
| Scaffold_8;HRSCAF_313 | 252,185,016 | G | A | 26,959.42 | G/G | A/A | DEM.v1.00021387.1 |
| Scaffold_8;HRSCAF_313 | 252,185,583 | C | T | 335.44 | C/C | T/T | DEM.v1.00021387.1 |
| Scaffold_8;HRSCAF_313 | 252,517,718 | G | A | 1,319.42 | G/G | A/A | DEM.v1.00021374.1 |
| Scaffold_8;HRSCAF_313 | 252,517,781 | C | T | 2,920.42 | C/C | T/T | DEM.v1.00021374.1 |
| Scaffold_8;HRSCAF_313 | 252,517,929 | G | A | 2,027.42 | G/G | A/A | DEM.v1.00021374.1 |
| Scaffold_8;HRSCAF_313 | 252,518,075 | G | A | 2,624.42 | G/G | A/A | DEM.v1.00021374.1 |
| Scaffold_8;HRSCAF_313 | 252,519,980 | C | CA | 4,184.38 | C/C | CA/CA | DEM.v1.00021374.1 |
| Scaffold_8;HRSCAF_313 | 252,520,021 | G | C | 5,525.42 | G/G | C/C | DEM.v1.00021374.1 |
| Scaffold_8;HRSCAF_313 | 252,520,892 | G | A | 7,895.42 | G/G | A/A | DEM.v1.00021373.1 |
| Scaffold_8;HRSCAF_313 | 252,524,505 | G | A | 9,070.42 | G/G | A/A | DEM.v1.00021373.1 |
| Scaffold_8;HRSCAF_313 | 252,524,788 | C | A | 8,069.42 | C/C | A/A | DEM.v1.00021373.1 |
| Scaffold_8;HRSCAF_313 | 252,525,011 | G | T | 5,764.42 | G/G | T/T | DEM.v1.00021373.1 |
| Scaffold_8;HRSCAF_313 | 252,527,982 | A | G | 202.26 | G/G | NA | DEM.v1.00021373.1 |
| Scaffold_8;HRSCAF_313 | 252,533,266 | A | G | 7,611.42 | G/G | A/A | DEM.v1.00021372.1 |
| Scaffold_8;HRSCAF_313 | 252,533,488 | A | C | 8,740.42 | C/C | A/A | DEM.v1.00021372.1 |
| Scaffold_8;HRSCAF_313 | 252,536,776 | T | G | 3,696.42 | T/T | G/G | DEM.v1.00021372.1 |
| Scaffold_8;HRSCAF_313 | 252,537,453 | T | G | 7,249.42 | G/G | T/T | DEM.v1.00021372.1 |
| Scaffold_8;HRSCAF_313 | 252,537,798 | A | C | 5,025.42 | C/C | A/A | DEM.v1.00021372.1 |
| Scaffold_8;HRSCAF_313 | 253,007,304 | A | G | 284.21 | NA | G/G | DEM.v1.00021360.1 |
| Scaffold_8;HRSCAF_313 | 253,007,361 | C | A | 208.21 | NA | A/A | DEM.v1.00021360.1 |
| Scaffold_8;HRSCAF_313 | 253,546,888 | C | CTTT | 87 | C/C | C/CTTT | DEM.v1.00021337.1 |
| Scaffold_8;HRSCAF_313 | 253,551,357 | G | GT | 59.82 | G/GT | G/G | DEM.v1.00021336.1 |
| Scaffold_8;HRSCAF_313 | 253,556,897 | T | C | 52.44 | T/T | T/C | DEM.v1.00021335.1 |
| Scaffold_8;HRSCAF_313 | 253,787,702 | C | CA | 205.15 | C/CA | C/C | DEM.v1.00021323.1 |
| Scaffold_8;HRSCAF_313 | 254,223,794 | C | T | 43.2 | NA | C/T | DEM.v1.00021274.1 |
| Scaffold_8;HRSCAF_313 | 254,223,821 | G | C | 51.2 | NA | G/C | DEM.v1.00021274.1 |
| Scaffold_8;HRSCAF_313 | 254,223,822 | G | A | 51.2 | NA | G/A | DEM.v1.00021274.1 |
| Scaffold_8;HRSCAF_313 | 254,223,890 | A | G | 64.17 | NA | G/G | DEM.v1.00021274.1 |
| Scaffold_8;HRSCAF_313 | 254,264,224 | A | C | 57.86 | A/A | C/C | DEM.v1.00021266.1 |
| Scaffold_8;HRSCAF_313 | 254,264,225 | G | C | 57.86 | G/G | C/C | DEM.v1.00021266.1 |
| Scaffold_8;HRSCAF_313 | 254,264,229 | A | T | 57.86 | A/A | T/T | DEM.v1.00021266.1 |
| Scaffold_8;HRSCAF_313 | 254,264,232 | G | A | 57.86 | G/G | A/A | DEM.v1.00021266.1 |
| Scaffold_8;HRSCAF_313 | 254,271,719 | CA | C | 68.2 | CA/CA | CA/C | DEM.v1.00021265.1 |
| Scaffold_8;HRSCAF_313 | 254,271,763 | C | T | 1,495.42 | C/C | T/T | DEM.v1.00021265.1 |
| Scaffold_8;HRSCAF_313 | 254,271,771 | G | A | 1,629.42 | G/G | A/A | DEM.v1.00021265.1 |
| Scaffold_8;HRSCAF_313 | 254,273,130 | T | C | 5,225.42 | T/T | C/C | DEM.v1.00021265.1 |
| Scaffold_8;HRSCAF_313 | 254,276,266 | T | C | 5,432.42 | T/T | C/C | DEM.v1.00021265.1 |
| Scaffold_8;HRSCAF_313 | 254,276,272 | T | C | 5,509.42 | T/T | C/C | DEM.v1.00021265.1 |
| Scaffold_8;HRSCAF_313 | 254,276,752 | C | T | 5,925.42 | C/C | T/T | DEM.v1.00021265.1 |
| Scaffold_8;HRSCAF_313 | 254,276,757 | A | G | 5,950.42 | A/A | G/G | DEM.v1.00021265.1 |
| Scaffold_8;HRSCAF_313 | 254,277,207 | G | A | 3,717.42 | G/G | A/A | DEM.v1.00021265.1 |
| Scaffold_8;HRSCAF_313 | 254,310,720 | C | G | 757.42 | G/G | C/C | DEM.v1.00021264.1 |
| Scaffold_8;HRSCAF_313 | 254,310,735 | T | C | 48.19 | T/T | T/C | DEM.v1.00021264.1 |
| Scaffold_8;HRSCAF_313 | 254,312,276 | A | G | 20,333.42 | G/G | A/A | DEM.v1.00021264.1 |

Chr., chromosome; Ref., reference; Alt., alternative; R, resistant; S, susceptible.
